# Outcomes of heart failure with reduced, mildly reduced, or preserved ejection fraction: the ESC HF III registry

**DOI:** 10.1093/eurheartj/ehaf1074

**Published:** 2026-02-25

**Authors:** Lars H Lund, Aldo P Maggioni, Maria G Crespo-Leiro, Cecile Laroche, Israel Gotsman, Belma Pojskic, Eleonora B Vataman, Lucica Grigorica, Hamayak Sisakian, Duska Glavas, Dulce A Brito, Stefan Anker, Ovidiu Chioncel, Gerasimos Filippatos, Mitja Lainscak, Theresa A McDonagh, Alexandre Mebazaa, Massimo Piepoli, Frank Ruschitzka, Gianluigi Savarese, Petar M Seferović, Marco Metra, Giuseppe Rosano, the ESC EORP HF III National Leaders and Investigators, A Vahanian, A Budaj, N Dagres, N Danchin, V Delgado, J Emberson, O Friberg, C P Gale, G Heyndrickx, B Iung, S James, A P Kappetein, A P Maggioni, N Maniadakis, K V Nagy, G Parati, A-S Petronio, M Pietila, E Prescott, F Ruschitzka, F Van de Werf, F Weidinger, U Zeymer, C P Gale, B Beleslin, A Budaj, O Chioncel, N Dagres, N Danchin, J Emberson, D Erlinge, M Glikson, A Gray, M Kayikcioglu, A P Maggioni, K V Nagy, A Nedoshivin, A-P Petronio, J W Roos-Hesselink, L Wallentin, U Zeymer, B A Popescu, D Adlam, A L P Caforio, D Capodanno, Ovidiu Chioncel, M Dweck, D Erlinge, Laurent Fauchier, Marek Gierlotka, M Glikson, Tina Hansen, J Hausleiter, B Iung, M Kayikcioglu, P Ludman, L H Lund, A P Maggioni, Julien Magne, S Matskeplishvili, B Meder, Julinda Mehilli, K V Nagy, A Nedoshivin, D Neglia, A A Pasquet, Eva Prescott, J W Roos-Hesselink, F J Rossello, S M Shaheen, A Torbica, B Iung, B Popescu, D Adlam, C Bouleti, A Caforio, D Capodanno, L Fauchier, M Gilard, S James, P Ludman, J Magne, A Pasquet, T Pilgrim, J Rossello, S Shaheen, AvTimoteo, A Torbica, U Zeymer, Marisa Crespo-Leiro, Petar Seferovic, Frank Ruschitzka, Gerasimos Filippatos, Alexandre Mebazaa, Massimo Piepoli, Andrew Coats, Stefan Anker, Theresa McDonagh, Mitja Lainscak, Giuseppe Rosano, Aldo Maggioni, Lars Lund, Ahmed Bennis, Andrejs Erglis, Andrzej Gackowski, Alena Kurlianskaya, Amina Rakisheva, Alex Simms, Barnabas Gellen, Bela Merkely, Candida Fonseca, Daniela Cassar Demarco, Duska Glavas, Eva Goncalvesova, Eleonora Vataman, Elizabeta Srbinovska Kostovska, Erkin Mirrakhimov, Gani Bajraktari, Grigorios Giamouzis, Artan Goda, Gulnaz Dadashova, Hamayak Sisakian, Hadi Skouri, Heli Tolppanen, Israel Gotsman, Jean Beissel, Jelena Celutkiene, Jose Manuel Garcia Pinilla, Larisa Dizdarevic-Hudic, Lars Lysgaard Gullestad, Leonid Voronkov, Magdy Abdelhamid, Micha T Maeder, Mitja Lainscak, Morten Schou, Massimo Francesco Piepoli, Marija Polovina, Milos Taborsky, Mikheil Tsverava, Naima Hammoudi, Ovidiu Chioncel, Petra Van Pol, Plamen Gatzov, Belma Pojskic, Petar M Seferovic, Rudolf Berger, Stefan Stoerk, Timur Abdullaev, Tiina Uuetoa, Vassilis Barberis, Vyacheslav Mareev, Wachter Rolf, Walter Droogne, Yuksel Cavusoglu, Zumreta Kušljugić, S Benkhedda, D Djermane, M Baouni, F Benouareth, K Mouzaoui, N Dahimene, S Mansouri, F Kerkouri, A Chibane, M Djouhri, S Benferhat, L Talbi, Y Bouhouita-Guermech, R Benkouar, A Boudrifa, H Elnaajer, K Bouasria, O Kassoul, Y Tir, D-E Nibouche, A Sik, A Bounah, N Hammoudi, G Sofiane, M Bouame, A Sayah, E Tebbache, A Kachenoura, F Daimellah, M T C Bouafia, M Bouafia, N Dammene Debbih, W Takdemt, T Manukyan, L Tumasyan, A Chilingaryan, A Stepanyan, L Tunyan, H Hayrapetyan, K Azaryan, M Tadevosyan, H Poghosyan, H Sisakian, G Martirosyan, L Sahakyan, M Hovhannisyan, S Pepoyan, A Kurlianskaya, D Salauyou, A Kozyrava, O Shatova, T Troyanova-Shchutskaia, M Vanderheyden, A Moya, H Batjoens, A-C Pouleur, O Gurne, E Tshilanda, F Severino, W Mullens, M Dupont, S Christiaens, C Claes, S Lenaerts, D Derthoo, M Dumoulein, S Naessens, I Senesael, M De Coninck, W Droogne, E Sels, V Servaes, P Troisfontaines, E Hoffer, M Melissopoulou, D Malmendier, M Massoz, S Jacquet, V Dinraths, E Begic, A Mujakovic, A Subo, A Selimagic, F Custovic, D Mackic, E Dzambasovic-Karaselimovic, A Cehajic, A Djozic, A Biscevic-Obradovic, F Kadic, E Hrvat, I Kurbasic, M Tuce, A Durak-Nalbantic, A Dzubur, E Jahic, S Sokolovic, Z Gljiva Gogic, K Aganovic, F Zvizdic, A Begic, N Resic, E Hodzic, M Halilcevic, M Vucijak Grgurevic, N Hadžibegic, N Sabanovic Bajramovic, S Miseljic, E Smajic, Z Kušljugic, L Dizdarevic-Hudic, K Kovacevic, D Loncar, M Kovacevic, M Selimovic, U Pajic, I Hudic, A Avdic, A Bijedic, A Brkic, D Mršic, E Becirovic, I Bijedic, B Pojskic, J Djelmic, M Ejubovic, L Pojskic, E Ramic, M Sammak, H Selimovic, E Stimjanin, M Sut, H Torlak, I Bisco, D Bogicevic, A Brkovic, R Salgado, A Silva, B Azeredo, D De Souza, I Couri, L Salgado, M Tokmakova, D Jovanovska, N Runev, B Stoimenov, E Manov, R Pancheva, V Kolev, V Mincheva, V Tsanova, O Eftimova, I Gruev, M Mintchev, V Stoyanovski, T Petrusheva, V Persic, M Barisic, D Raljevic, D Glavas, D Milicic, J A Borovac, T Zaninovic Jurjevic, J Sikic, V Persic, K Pesek, K Pesek, S Roginic, N Borsic, J Sikic, T Friscic, Z Planinic, T Christodoulides, S Kakoulli, V Barberis, T Michaelidou, H Hasan-Ali, M Abdel Ghany, A Halim, S Dimitry, P P S Selwanos, M Hassan, A-S M Sabry, Y A Abdelhady Ahmed, T Osman, M Eshak, M El Sayed, K Menyawi, A Roshdy, W El Kilany, A Abdeltawab, A R El Sayed, O Botrous, A El Bagoury, A Samir, H Kandil, M Hosny, M Abdelghany, M Abdelhamid, M K Shokry, S Essam, A Kamal, H Ammar, A Kamel, A Magdy, H Elnesr, M Amin, M M Yousif, A H Eladawy, M F Areed, M H Abdelnabi, A G Mahmoud, M F Abdelrahman, A Elbahry, A Saad, M Ali, T Uuetoa, S Udalova, T Anier, S Viks, B Veermäe, H Tolppanen, K Oksaharju, N Mewton, A Jobbé Duval, C Boiteux, C Bergerot, G Baudry, L Sebbag, M Buisson, D Mohamed-Said, F El Harrane, M Koenig, N El-Jonhy, A Galat, D Bodez, C Chalard, F Djelaili, S Guendouz, S Oghina, T Damy, M Idjellidaine, M Kharoubi, N Djefel, Y Sellah, R Pilato, J Inamo, A Monfort, N Ozier-Lafontaine, D Guijarro, T Fourme, A Monard, P Bouheret, M Salvat, A Boignard, C Augier, C Casset, M Maurin, E Plan, D Pollet, P Jourdain, E Berthelot, M T Bailly, N Hrynchyshyn, G Leprun, B Livarek, J-L Georges, N Baron, C Charbonnel, J-B Azowa, C Bachelet, P Cloitre, P Poret, S Braun, O Zid, G Kabalu, A Denizet Mulocher, C Bros, C Dericbourg, C Saint Andre, E Lefebvre, J-F Laine, G Terrien, J-C Amirault, D Lecuyer, M Rousseau, F Beauvais, A Cohen-Solal, D Logeart, N Bennacer, B Gellen, J-M Tartiere, F Challal, F Fellini, R Landes, A Elkeurti, C Genin, P Armangau, V Gardan, M Galinier, O Lairez, J Roncalli, C Biendel, C Delmas, C Delon, E Cariou, P Fournier, R Itier, A Karavidas, D Balta, C Mandila, E Velaoras, I N Karavidas, L Michalis, K Naka, A Bechlioulis, L Lakkas, A Rammos, I Dimou, C Karvounis, M-A Bazbani, B Merkely, A Kosztin, N Nyolczas, D Vagany, I Juhasz, S Papp, M Szabo, E Szogi, A Pálinkás, I Szoko Csaszar, I Juhasz Nagy, S Rostásné Toth, T Habon, M Rabai, R Halmosi, R Gal, L Illes, S Kovacsne Levang, H Ali Farhan, I F Yaseen, E Radzishevsky, I Gotsman, O Ezra, T Levinas, I Nordkin, S Benyamin, Tel M Laufer-Perel, A Milwidsky, B Sadeh, D Wexler, O Havakuk, Y Arbel, M Revivo, S Sadon, M Metra, L Italia, L Rossi, Cassano Delle A Passantino, R Carbonara, C Rizzo, R La Gioia, M Milli, F Grossi, N D Brunetti, A Mallardi, M Correale, M Penco, G Patti, S Romano, R Petroni, E Salustri, S Minardi, S Scalvini, E Zanelli, A Olivares, P Agostoni, M Doldi, E Salvioni, P Gugliandolo, A Frisinghelli, C Calloni, C Cadeddu Dessalvi, M Deidda, A Peccianti, D Cocco, E Garau, A Cittadini, A Salzano, F Giallauria, G Crisci, R Lucci, A M Marra, V Valente, R D´assante, P Perrone-Filardi, P Gargiulo, S Paolillo, L Bardi, S Dellaversana, G Novo, A Ferlisi, D Adorno, E Bonni, E Corrado, F Sabatino, S Novo, M F Piepoli, B Matrone, G Halasz, L Moderato, V Pelizzoni, M Mancone, C Miotti, E Pagliaroli, M Magnocavallo, San Vito Al D Pavan, T Durat, V D’Onofrio, A Gardin, G Ganci, F Martinis, N Pezzutto, S Maier, G Sinagra, M Merlo, L Restivo, F Ramani, D Miani, M Baldassi, M Driussi, F L Ribichini, L Maritan, M Cicoira, M Lia, M Setti, M De Stefano, B Zholdin, G Mamedova, R Iznatova, Y Sim, R Tuleutayev, A Nurmukhambetova, I Yakupova, D Urazbekov, N Aidargaliyeva, A Akanova, A Makhmudova, A Tleules, G Nurbekova, M Akilbekova, S Nurlybai, A Aizhan, A Rakisheva, A Illyassova, A Dzholdasbekova, Z Ismagulova, A Kassenova, M Alkenova, A Aizhan, E Mirrakhimov, K Neronova, J Esenbekova, D Zaliaduonyte, J Laukaitiene, J Borkyte, A Mazutaviciute, R Norvilaite, J Celutkiene, E Paleviciute, J Simonavicius, L Gedvilaite, M Laukyte, E Lycholip, T Simbelyte, R G Xuereb, S Xuereb, W Camilleri, M Farrugia, J Fleri Soler, L-L Buttigieg, R Xuereb, A M Moore, D Cassar Demarco, C Attard, M Vella, I Grech, R Bonnici, R Buhagiar, G Buhagiar, T Farrugia, M O De Los Rios Ibarra, A Z Baños Velasco, F M Vizcaíno Rios, A Mendez, A Alvarez, C Guizar, S Leon Gonzalez, L Resendiz-Barron, E Vataman, D Bursacovschi, D Lisii, E Paraniuc, J Cazacu, M Dogot, N Botnari, L Grib, S Filimon, E Samohvalov, L Purteanu, A Grivenco, V Revenco, I Cabac-Pogorevici, I Cojuhari, A Bennis, S Chraibi, A El Makhlouf, S Ferhi, S Soulami, N Mouine, A Moustaghfir, K Adnan, C Bencheqroun, G Abidi, H Elmousalami, S Hajib, L Bouzoubaa, M Ghita, I Nassiri, S Abderrazak, Z Benchaouia, G Benhayoune, N Boughaidi, D Ghellab, A Sourat, B Menebhi, M Bensaoud, A El Ouazzani, A Lachhab, A Zakaria, F El Idrani, H Lachhab, N Elouafi, A Hbali, G Elmazani, I Alla, S Ztot, M Najat, B E El Younassi, I Asfalou, L Chami, M Raissouni, S Abdelali, A Benthami, A Drissi Kacemi, S Essadki, A Louali, K Bennis, Y Lididi, R Bouhouch, R Mesbahi, S Bouzidi Belmajdoub, I Ukpeh, R Basake, E-O Akpan, E Emmanuel, O Mbang, North M Gjerakaroska Radovikj, E Srbinovska Kostovska, I Mitevska Peovska, L Poposka, G Hogalmen, M German, L Lysgaard Gullestad, C Holt Bendz, O Haugene, E Bjorkelund, K Mizia-Stec, K Bula, J Nessler, A Gackowski, A Siniarski, M Kabat, P Rubis, A Karabinowska, E Dziewiecka, S Wisniowska-Smialek, M Lelonek, A Cieslak, A Klaus, J Szulc, K Drazek, K A Nguyen, M Kiedrowska, A Bielecka-Dabrowa, M Pyziak Stepien, M Rybak, P Leczycki, P Chrusciel, A Wittczak, M Banach, A Chuda, A Szwedzinska, M Rembek Wieliczko, D Mrozowska, F Pawliczak, J Lewek, M Maciejewski, A Cichocka-Radwan, A Bikiewicz, K Janikowski, J Szponar, A Kujawa, A Sutkowska, B Cebulak, K Pirog, J Wieczorek, M Suchecka, S Goliszek, M Gierlotka, E Cierpiala, J Bugajski, J Plonka, K Rekucki, E Straburzynska-Migaj, D Budzynski, M Gasiorowski, M Dudek, G Skonieczny, A Dolacinska, A Metzgier Gumiela, A Stawicka, M Rolirad, A Kaplon-Cieslicka, M Wawrzacz, K Ozieranski, M Kleszczewska, A Tyminska, R J Gil, A Pawlak, M Wozniak, M Pietraszek, A Nazaruk, A Bobel, A Smolarczyk, D Wiligorska, D Ziecik, J Latek, K Byczkowska, P Leszek, J Urbanowski, N Wiligorska, Z Kalarus, T Kukulski, A Swiatkowski, M Szulik, N Kandora, Zielona P Jesionowski, T Zemleduch, A Kasperowicz, K Sosinska, A Camacho, R Fernandes, H Costa, C Tavares Aguiar, A Tralhão, A Ventosa, C Brízido, C Strong, B Rocha, G Cunha, D Brito, A Nunes-Ferreira, I Aguiar Ricardo, R Santos, S Pereira, T Rodrigues, J Agostinho, J Brito, J Rigueira, N Cunha, P António, P Morais, D Cravo, C Fonseca, C Rodrigues, I Araujo, J Presume, M Proenca, S Maltes, B Moura, E Barreira, P Janeira, C Ferrao, J Silva-Cardoso, A Sousa, B Mena, X Resende, M Fonseca, M Braga, M Campelo, E Moreira, P Araújo, P Diogo, R Pinto, S Amorim, B Moura, Baia C Pop, A Iosip, C-J Sinescu, N Avram, D G Baltag, N Samoila, M Dorobantu, A Scafa Udriste, A Scarlatescu, N Oprescu, O Fronea Gheorghe, O Chioncel, L Antohi, O Geavlete, G-A Dan, I-C Daha, S Bari, C Delcea, D C Ciuculete, I Lupasteanu, C Stanescu, A Vijan, C Militaru, A Giuca, G C Moise, E G Pascu, L Grigorica, C Corciova, Targu T Benedek, M Chitu, N Rat, R Hodas, D Opincariu, I S Benedek, D Lighezan, A C Florea, R Buzas, V Ivan, Russian A Molchanova, M Piskareva, E Bulgakova, E Kudryashova, A Galyavich, L Baleeva, Z Galeeva, A Fendrikova, V Skibitskiy, Z Sokaeva, V Babayan, N Spiropulos, E V Resnik, A I Kovaleva, M S Komissarova, V A Lazarev, V Larina, Y Belenkov, Y Danilogorskaya, E Zheleznykh, E Privalova, I Ilgisonis, V Kaplunova, M Kozhevnikova, G Shakaryants, A Shchendrygina, A Yusupova, V Zektser, Z Kobalava, E Shavarova, L Karapetyan, G Gendlin, A Melekhov, I Zakharova, M Kuznetsova, M Yunyaeva, G Arutyunov, A Arutyunov, R Muradyan, S Golitsyn, E Gupalo, N Mironova, O Drapkina, R Myasnikov, T Pavlunina, V Dindikova, Y Mareev, E Andreenko, K Krupychka, M Kudryavtseva, O Kulikova, I Orlova, J Begrambekova, Nizhny N Vinogradova, I Fomin, N Koziolova, A Veclich, P Karavaev, A Chesnikova, O Kolomatskaya, V Gavrina, E Smirnova, A Karakiyan, I Budanova, M Sitnikova, A Kuular, M Trukshina, T Lelyavina, D Duplyakov, E Zorina, R Chernitsov, A Sergeeva, A Garganeeva, O Tukish, K Kopeva, V Aleksandrenko, A Surminova, A Tsareva, T Musurok, A Garkusha, A D Ristic, B Ivanovic, D Simic, M Ašanin, P M Seferovic, J Simic, V Kovacevic, G Krljanac, D Matic, M Mihailovic, M Ostojic, M Polovina, M Tesic, D Djikic, D Simeunovic, I Petrovic Djordjevic, I Veljic, I Milinkovic, K Andjelkovic, A Uscumlic, D Sacic, M Dekleva Manojlovic, S Veljkovic, D Stefanovic, J Stevic, S Hinic, M Zdravkovic, A Dokovic, J Djokic, V Mudrenovic, V Popadic, S Klasnja, S Radovanovic, M Radovanovic, V Celic, A Ilic, N Blagojevic, D Bosnjakovic, D Toncev, Niš: S Apostolovic, D Stanojevic, S Milutinovic, Niska B Ilic, M Deljanin Ilic, L Nikolic, M Stojanovic, D Petrovic, D Simonovic, S Šaric, D Hristov, Sremska I Srdanovic, J Dejanovic, T Popov, S Cemerlic Maksimovic, S Dimic, S Keca, V Drljavic, D Bogdanovic, I Popov, J Pavic Poljak, E Goncalvesova, M Luknár, P Solik, M Pytliak, P Bojcík, Murska M Lainscak, N Cmor, E Dora, L Majc Hodoscek, A Vogrincic Cernezel, B Leskovar, T Furlan, A Milanovic, K Vrbinc Vrtek, S Poznic, V Grilj, M Režun, V Martinez Mateo, M J Fernandez Anguita, C Ortiz Cortés, A Valle Muñoz, H Morillas Climent, J Seller Moya, S Darnés Soler, S Cudini, S López Fernández, L Jordán Martínez, F Bermúdez Jiménez, La M Crespo Leiro, D Couto Mallon, E Barge Caballero, G Barge Caballero, M J Paniagua Martin, P Pardo Martinez, C Naya Leira, C Riveiro Rodriguez, M Martinez Castro, P Blanco Canosa, Z Grille Cancela, J M Garcia Pinilla, A Robles Mezcua, A Rodriguez Cordoba, C Cruzado Alvarez, L Morcillo Hidalgo, P Marquez Camas, A I Perez Cabeza, P Redondo-Gomez, M Robles Mezcua, J L Bonilla Palomas, L Lund, M Nygren, G Savarese, C Hage, E Jonsson, E Ottenblad, F Granstrom, H Lundberg, K Karlsson, Syrian Arab Y Bani Marjeh, A Abdin, F Alhussein, F Mgazeel, F Yavuz, A Karakus, Alanya A Coner, S Akinci, B Demirkan, O Akkus, A Genc, F O Arican Ozluk, H Harbalioglu, Y Cavusoglu, E Babayigit, E Sener, E I Yuce, H Altay, Ö Yildirimtürk, C Altin, B Kilicaslan, B Unal, H Acet, N Cetin, C Burak, D Karacimen, A Agacdiken Agir, Y U Celikyurt, A Celik, E E Sahin, O Sakarya, M Demir, O Basaran, A E Atas, O Khaniukov, I Vakaliuk, I Drapchak, V Sovtus, N Tymochko, O Prytuliak, V Tseluyko, N Matviichuk, M Kopytsya, T Storozhenko, I Rudyk, O Medentseva, D Babichev, L Voronkov, A Liashenko, I Rudenko, K Lazareva, N Sishkina, A Honcharuk, O Vasylenko, Y Antoniuk, M Dolzhenko, L Hrubyak, L Lobach, T Simagina, S Kozhuhov, N Dovganych, N Thor, M Danko, O Yarynkina, O Bazyka, A Parkhomenko, A Stepura, D Bilyi, O Irkin, O Dovhan, V Batushkin, D Poddyachaya, O Zharinov, B Todurov, I Lischuk, K Rudenko, V Zhebel, I Pashkova, L Sursaieva, Krivoy V Potabashniy, V Fesenko, O Markova, O Kniazieva, O Berezin, O Kremzer, United M Aldwaik, A Bolger, R Manley, V Garvey, T Abdullaev, S Mirzarakhimova, A Rasulov, A Karimov, H Gulomov, I Tsoy, R Kurbanova, R Bekbulatova, U Kamilova, D Tagaeva

**Affiliations:** Department of Cardiology, Karolinska Institutet, Norrbacka, S1:02, 17176 Stockholm, Sweden; ANMCO Research Center, Heart Care Foundation, Florence, Italy; Department of Cardiology, Hospital Universitario A Coruña (CHUAC), Cardiology, INIBIC (Institute Investigacion Biomedica A Coruña), A Coruña, Spain; Department of Cardiology, CIBERCV, A Coruna, Spain; Department of Cardiology, Universidad de A Coruña (UDC), A Coruna, Spain; European Society of Cardiology, Sophia-Antipolis, France; Hadassah University Hospital, Jerusalem, Israel; Cantonal Hospital Zenica, Medical Faculty University of Zenica, Zenica, Bosnia and Herzegovina; Heart Failure and Cardiac Rehabilitation, Institute of Cardiology, Chisinau, Republic of Moldova; Department of Cardiology, Emergency Hospital ‘SF Apostol Andrei’ Galati, Galati, Romania; Head of Clinic of General and Invasive Cardiology, University Hospital 1, Yerevan State Medical University, Yerevan, Armenia; Department of Cardiovascular Disease, Intensive Care Unit, University Hospital Split, Split, Croatia; Heart and Vessels, Cardiology, ULS Santa Maria, Hospital Universitário, Lisboa, Portugal; Centro Cardiovascular da Universidade de Lisboa, CCUL@RISE, Faculdade de Medicina, Universidade de Lisboa, Lisboa, Portugal; Department of Cardiology (CVK) of German Heart Center Charité, German Centre for Cardiovascular Research (DZHK) Partner Site Berlin, Charité Universitätsmedizin, Berlin, Germany; Department of Cardiology, University of Medicine Carol Davila, Bucharest, Romania; Department of Cardiology, Athens University Hospital Attikon, School of Medicine, National and Kapodistrian University of Athens, Athens, Greece; Faculty of Medicine, University of Ljubljana, Ljubljana, Slovenia; Division of Cardiology, General Hospital Murska Sobota, Murska Sobota, Slovenia; Department of Cardiology, King's College Hospital, London, UK; Université Paris Cité, MASCOT Inserm Unit, Hôpitaux Universitaires Saint Louis et Lariboisière, APHP, Paris, France; University Cardiology, IRCCS Policlinico S Donato, Via Morandi 30, 20130 San Donato Milanese, Milan, Italy; Department of Cardiology, University Heart Center, University Hospital Zurich and University of Zurich, Zurich, Switzerland; Center for Translational and Experimental Cardiology (CTEC), Department of Cardiology, University Hospital Zurich, University of Zurich, Zurich, Switzerland; Department of Clinical Science and Education, Södersjukjuset, Karolinska Institutet, Stockholm, Sweden; Serbian Academy of Sciences and Arts, Belgrade, Serbia; Medical and Surgical Specialties, Radiological Sciences and Public Health, University of Brescia, Cardiology, Brescia, Italy; Department of Human Sciences and Promotion of Quality of Life, San Raffaele Open University of Rome, Rome, Italy; Department of Cardiology, San Raffaele Cassino Hospital, Cassino, Italy

**Keywords:** Acute heart failure, Chronic heart failure, Ejection fraction, Registry, Outcomes, Event rates, Trial design

## Abstract

**Background and Aims:**

To assess in-hospital and 1-year cause-specific outcomes in the contemporary European Society of Cardiology (ESC) Heart Failure (HF) III Registry.

**Methods:**

Patients were enrolled in European or ESC affiliated countries and characterized in detail regarding clinical characteristics and cause-specific outcomes.

**Results:**

Between 1 November 2018 and 31 December 2020, 10,162 patients were enrolled from 220 centres in 41 countries. Of these, 39% had acute HF (‘AHF’, age 70 [62–79] years, 36% women) and 61% had out-patient visit for HF [‘out-patient HF’, age 66 (58–75) years, 33% women]. Overall, 58% had HF with reduced ejection fraction (HFrEF), 17% HF with mildly reduced ejection fraction (HFmrEF), and 25% HF with preserved ejection fraction (HFpEF). In AHF, median [interquartile range (IQR)] duration of hospitalization was 9 (6–14) days, and 5.1% died in hospital (HFrEF 5.2%; HFmrEF 4.8%, HFpEF 3.4%). In AHF discharged alive and in out-patient HF, after a median (IQR) follow-up of 376 (360–432) days, all-cause, cardiovascular (CV), and unknown-cause mortality rates per 100 patient-years were as follows: AHF HFrEF: 19, 13, and 3.0 per 100 patient-years. The corresponding numbers were in AHF HFmrEF: 22, 11, and 6.3; AHF HFpEF: 16, 7.0, and 4.7; out-patient HFrEF: 6.6, 4.3, and 0.9; out-patient HFmrEF: 4.0, 2.6, and 0.8; out-patient HFpEF: 3.9, 1.7, and 1.2. At least one (re-)hospitalization for HF was experienced in 44% AHF HFrEF, 42% AHF HFmrEF, 36% AHF HFpEF, 21% out-patient HFrEF, 14% out-patient HFmrEF, and 18% out-patient HFpEF.

**Conclusions:**

In HF in Europe and affiliated countries, in-hospital mortality was 5.1% and greater with lower ejection fraction. Among hospital survivors and out-patients over 1 year of follow-up, event rates per 100 patient-years varied for death, 3.9–22, CV death 1.7–13, and unknown cause of death 0.8–6.3. The percent of patients that were (re-)hospitalized for HF at least once over 1-year follow-up ranged 14–44% and was twice as high post-AHF compared with post-out-patient visit.

## Structured Graphical abstract

**Structured Graphical abstract:**
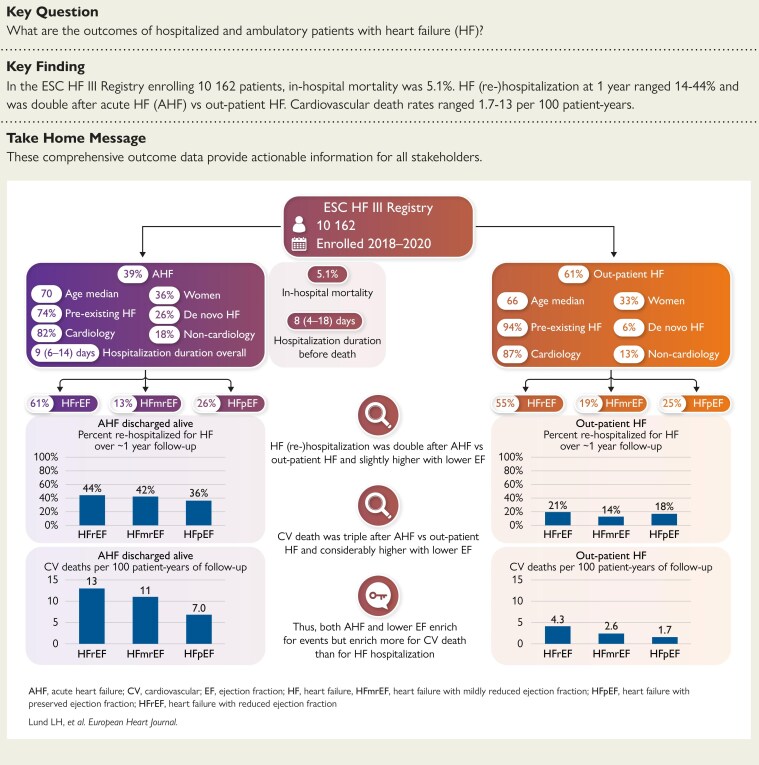

**See the editorial comment for this article ‘Europe's heart failure landscape: the persistent under-recognition of HFpEF’, by B. Moura *et al.*, https://doi.org/10.1093/eurheartj/ehaf981.**

## Introduction

Heart failure (HF) affects more than 64 million people worldwide and is increasing in prevalence, likely due to an ageing population, continuously improving treatment and survival after myocardial infarction, and improved recognition of HF with preserved ejection fraction (HFpEF).^1,2^ HFpEF in particular is expected to increase considerably in the future.^3^ Mortality and risk of HF hospitalization remain high and quality of life and functional capacity are poor.^1,4,5^

There are now extensive effective therapies for HF with reduced ejection fraction (HFrEF), suggestions that many of these may be effective in HF with mildly reduced ejection fraction (HFmrEF),^3^ but so far only sodium-glucose co-transporter 2/1 (SGLT2/1) inhibitors and mineralocorticoid receptor antagonists appear effective across the ejection fraction (EF) spectrum.^6,7^ In HFpEF, exercise and caloric restriction^8^ and glucagon-like peptide-1 agonists in obesity with concurrent HFpEF^9,10^ may be effective. Historically, patients hospitalized with acute HF (AHF) were excluded from trials of chronic HF treatment, but increasingly patients who are stable in the hospital are allowed into chronic HF trials.

There are numerous differences in characteristics and outcomes among patients with HF with different EF categories, and among patients with new-onset vs pre-existing and AHF vs out-patient HF, and in AHF, in-hospital vs post-discharge outcomes. These differences are critical to understand for clinical decision making, triage and prioritization, and for interpretation and design of clinical interventional trials. These categories have been studied individually in different registry, cohort, and trial dataset contexts, but these studies have been limited by small size, data granularity, or generalizability, and these HF categories and settings have rarely been studied together and thus there are no large generalizable granular direct comparisons between both different EF categories and in-patient vs out-patient settings. The European Society of Cardiology (ESC) HF III Registry aims to provide a large comprehensive analysis of HF in European and ESC-affiliated countries. The rationale and design,^11^ and the first co-primary analysis from ESC HF III Registry [which provided a comprehensive analysis of baseline HF characteristics and guideline-directed medical therapy (GDMT) treatment decisions]^12^ have been published. Here we provide the second co-primary analysis: In-hospital outcomes in AHF and 1-year outcomes in out-patient and post-AHF, and according to EF category.

## Methods

### Rationale and design

The ESC HF III Registry has been previously described.^11^ It is a prospective, international, multicentre, observational registry study of patients with HF. It includes patients regardless of EF, with either chronic or *de novo* HF who present with a non-urgent out-patient visit or with urgent worsening HF for care in the hospitalized, emergency department, or out-patient urgent care settings, and treated with intravenous medications (diuretics, vasodilators, vasopressors, or inotropes) for HF. The objectives include providing a comprehensive data set for both discovery and implementation science. Extensive baseline data are collected. The first co-primary analysis focused on baseline use of and decision making regarding GDMT.^12^ Here, we present the second co-primary analysis: outcomes.

### Oversight

The ESC HF III Registry is sponsored by the ESC EURObservational Research Programme (EORP; https://www.escardio.org/Research/registries/eorp). The HF III Chairperson (LHL) wrote the protocol with input from the EORP Oversight Committee (Appendix 1) and from the HF III Executive Committee (Appendix 2). National coordinators (the Steering Committee, Appendix 3) coordinated national activities and liaised with the Chairman, the sponsor team at EORP, and local investigators (Appendix 4).

### Setting

The ESC HF III Registry enrolled patients with HF in European, Mediterranean and some non-European countries, affiliated with the ESC. The registry complies with the 1975 Declaration of Helsinki; the locally appointed ethics committees approved the research protocol, and informed consent was obtained from all patients. The target enrolment was 10 000 patients. Detailed data elements and time points have been described.^11^ Data were entered manually by investigators and/or coordinators into a registry-specific electronic case report form, managed by EORP. Data were validated by EORP, and out-of-range, missing or incomplete data were queried by EORP to local sites.

### Statistical analysis

Descriptive data are presented with *n* (%) and median [interquartile range (IQR)] and groups were compared using non-parametric statistics. Natriuretic peptides were measured as either B-type natriuretic peptide (BNP) or N-terminal pro-B-type natriuretic peptide (NT-proBNP), which are not numerically comparable; therefore, in multivariable analyses, these were matched by their percentile distribution and then pooled as one variable.

In patients with AHF, length of stay and the proportion who died in hospital were assessed. Among all patients, cumulative incidence of all-cause death from enrolment (defined as date of hospital admission in AHF and date of out-patient visit) was compared in AHF vs out-patient HF and in HFrEF vs HFmrEF vs HFpEF and depicted with Kaplan-Meier curves and compared with the log rank test. For the cumulative incidence of cardiovascular (CV) death, competing risk of death from other causes was taken into account and the Aalen-Johansen estimator was calculated and groups were compared with the Gray test. Unknown cause of death was counted as CV death and also reported separately. Univariable (unadjusted), age- and sex-adjusted, and multivariable (adjusted) Cox regressions and cumulative incidence function regressions were performed to assess the crude and independent association between AHF vs out-patient HF and between the three EF categories and all-cause mortality and CV mortality. The unadjusted hazard ratios (HRs) provide information about actual risk in the specific patient category. This might be useful from an epidemiological or trial design perspective. The adjusted HRs provide information about how much of the risk is explained by the specific category, rather than by associated confounders such as age and sex, and additionally other measured clinical confounders. This might be useful when seeking to understand the potential causal, mechanistic or biological contribution of a specific category to the risk of an outcome. In the age- and sex-adjusted Cox model and cumulative incidence function model, age was modelled as splines and age × sex interaction term was included. This interaction term was non-significant in all age- and sex-adjusted analyses. Therefore, in the fully adjusted models, the age × sex interaction term was not included. The multivariable Cox regressions were performed with adjustment for 14 clinically relevant covariates (listed in the legend to *Figure 2*). Natriuretic peptides, unlike the other covariates, were missing in a meaningful proportion of patients. Therefore, a sensitivity analysis was conducted excluding natriuretic peptides from the multivariable Cox regressions. For Cox regressions, assumption of proportional hazards was tested with the Schoenfeld residuals and for cumulative incidence function regressions, with calculation of interaction between covariable and time.

Outcomes were also assessed as event rates per 100 patient-years of follow-up and presented with 95% confidence intervals (CIs) using Poisson models. This was done unadjusted and adjusted for age and sex. The outcome dates were assessed for death but not for hospitalization. Therefore, the time to first hospitalization was not assessed. However, the total number of days of hospitalizations during the total number of days of follow-up was assessed.

Finally, 1-year mortality probability with 95% CIs were assessed in unadjusted, adjusted for age, sex and age × sex interaction, and fully adjusted models.

All analyses were performed using SAS statistical software version 9.4 (SAS Institute Inc, Cary, NC, USA) and R version 4.3.0

## Results

### Baseline characteristics

Between 1 November 2018 and 31 December 2020, 10,162 patients were enrolled from 220 centres in 41 countries. Among 10 162 patients, 32 patients (0.3%) had unknown information on setting of enrolment (AHF vs out-patient HF), leaving 10 130 patients for the AHF vs out-patient HF analyses, 39% enrolled with AHF and 61% as out-patient HF. EF was missing in 111 patients (1.1%), leaving 10 051 for EF category analyses and was categorized as reduced (HFrEF, 58%), mildly reduced (HFmrEF, 17%), or preserved (HFpEF 25%).

*Table 1* shows selected baseline characteristics (more details on baseline characteristics are available in the baseline manuscript.^12^) Compared with out-patient HF, patients with AHF were older, slightly more commonly women, and had lower EF, higher New York Heart Association (NYHA) class, higher blood pressure (except those who died in-hospital, who instead frequently had hypotension), distinctly higher heart rate, lower estimated glomerular filtration rate, approximately 2–4 fold higher natriuretic peptide levels, and more comorbidities. Each of the common HF signs and symptoms were present in a majority of patients with AHF (and even more commonly in those who died in hospital), but only in a minority of patients with out-patient HF. There was minimal missing data, on the order of 1% for most variables, but NT-proBNP was unavailable in 49% and BNP in 83%, suggesting at least one-third of patients did not have natriuretic peptides assessed.

**Table 1:** Baseline characteristics at enrolment

Total *n* = 10 130^a^	Died in hospital (*n* = 200/3913; 5.1%)	AHF with follow-up (*n* = 2294, 59%)	AHF lost to follow-up (*n* = 1419, 36%)	Out-patient with follow-up (*n* = 4597/6217, 74%)	Out-patient lost to follow-up (*n* = 1620, 26%)	Missing data (%)
EF category						1.1
HFrEF	68%	59%	63%	54%	61%	
HFmrEF	13%	13%	13%	21%	14%	
HFpEF	19%	28%	24%	25%	25%	
Age (years)	72 (63–82)	71 (62–79)	69 (61–78)	66 (58–75)	66 (57–75)	0.0
Females	29%	37%	35%	34%	31%	0.1
BMI (kg/m^2^)	26 (23–29)	27 (24–31)	28 (25–32)	28 (25–31)	27 (25–30)	4.4
NYHA class III-IV	92%	81%	77%	33%	28%	1.7
Primary underlying HF aetiology						2.0
Ischaemic	56%	49%	52%	51%	54%	
Dilated cardiomyopathy	11%	13%	13%	17%	18%	
Other	33%	37%	35%	32%	28%	
Medical history						
Myocardial Infarction	46%	37%	41%	37%	42%	0.5
Stroke/transient ischaemic attack	15%	8%	10%	8.8%	9.2%	0.5
Atrial fibrillation						0.6
Permanent/Persistent	32%	38%	36%	28%	26%	
Paroxysmal	16%	13%	13%	11%	12%	
Diabetes						0.7
Yes, non-insulin treated	22%	21%	22%	21%	20%	
Yes, insulin treated	20%	16%	16%	11%	11%	
Arterial Hypertension	73%	73%	71%	65%	65%	0.5
Peripheral vascular disease	18%	14%	14%	11%	12%	1.0
Venous Thromboembolism	6.1%	3.8%	3.6%	2.6%	2.7%	0.5
CRT	7.4%	4.8%	4.0%	8.9%	11%	1.5
ICD	8.5%	8.9%	5.8%	17%	15%	1.5
Non-cardiovascular conditions	40%	30%	34%	27%	24%	1.2
Chronic obstructive pulmonary disease	14%	15%	14%	11%	10%	1.2
Dialysis	3.6%	1.1%	1.5%	1.4%	1.2%	1.2
Hepatic dysfunction	8.2%	3.7%	6.0%	3.6%	2.2%	1.2
Current active cancer	6.2%	3.8%	2.9%	2.9%	1.8%	1.2
Depression	6.7%	4.8%	6.6%	5.6%	5.2%	1.2
Cognitive dysfunction	8.8%	4.2%	7.9%	1.9%	2.6%	1.2
Rheumatoid arthritis	2.1%	1.3%	1.1%	1.2%	1.2%	1.2
Sleep apnoea	2.6%	3.9%	4.6%	5.4%	3.8%	1.4
Signs, symptoms at presentation						
Systolic blood pressure (mmHg)	110 (90–135)	130 (110–150)	128 (110–145)	122 (110–139)	122 (110–140)	1.3
Systolic blood pressure ≤110 mmHg	55%	26%	30%	28%	29%	1.3
Diastolic blood pressure (mmHg)	70 (60–80)	80 (70–90)	80 (70–90)	75 (65–80)	75 (69–80)	1.5
Heart rate (bpm)	95 (76–110)	87 (74–101)	86 (72–100)	70 (63–80)	70 (65–80)	1.0
Pulmonary rales	84%	69%	64%	19%	16%	1.3
Peripheral oedema	68%	68%	60%	26%	23%	1.1
Dyspnea at rest	82%	67%	62%	14%	13%	1.0
Orthopnea	72%	57%	50%	14%	11%	1.4
Jugular venous pulse >6 cm	56%	36%	32%	12%	12%	5.2%
Laboratory at presentation						
Haemoglobin (g/dL)	11.9 (10.1–13.7)	13.0 (11.3–14.4)	12.8 (11.3–14.2)	13.4 (12.2–14.7)	13.4 (12.1–14.6)	9.8
eGFR (mL/min/1.73 m²)	43 (26–60)	55 (39–74)	55 (38–74)	66 (49–84)	64 (48–82)	10
Potassium (mmol/L)	4.3 (3.9–4.9)	4.3 (3.9–4.7)	4.3 4.0–4.7)	4.5 (4.2–4.8)	4.4 (4.1–4.8)	12
BNP (pg/mL)	1108 (760–3457)	860 (400–1945)	800 (255–1870)	400 (188–641)	432 (189–1035)	83
NT-proBNP (pg/mL)	9005 (2977–21 340)	3838 (1359–10 156)	3666 (1127–8970)	1072 (465–2604)	1221 (572–2929)	49
NT-proBNP >1000 pg/mL	93%	81%	78%	52%	58%	49

^a^ *n* enrolled = 10 162 but *n* = 32 unknown if enrolled as AHF or out-patient; therefore *n* = 10 130 in *Table 1*. Data are at hospital admission in AHF and at or most recent before out-patient visit. Data are median (interquartile range) or percent. AHF = acute heart failure; EF = ejection fraction; HFrEF = heart failure with reduced ejection fraction; HFmrEF = heart failure with mildly reduced ejection fraction; HFpEF = heart failure with preserved ejection fraction; IQR = interquartile range; BMI = body mass index; NYHA = New York Heart Association; CRT = cardiac resynchronization therapy; ICD = implantable cardioverter-defibrillator; eGFR = estimated glomerular filtration rate; BNP = B-type natriuretic peptide; NT-proBNP = N-terminal pro-B-type natriuretic peptide.

### Hospital course and in-hospital mortality (for patients enrolled with AHF)

**Figure 1:**
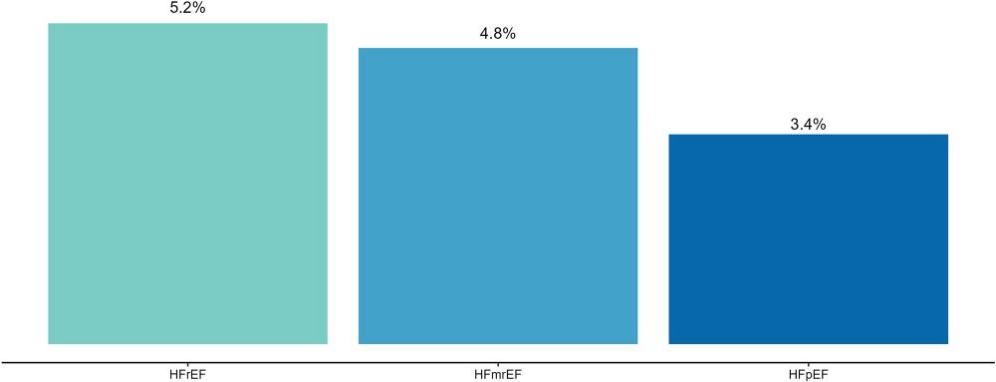
**AHF: In-hospital mortality rates.** Among patients enrolled with AHF (*n* = 3913, 39%), percent in-hospital mortality for HFrEF, HFmrEF, HFpEF. The overall in-hospital mortality was 5.1%. The duration of hospitalization (length of stay) was 9 (6–14) days overall, 8 (4–18) days in those who died during hospitalization, and 9 (6–13) days in those who were discharged alive

Among patients with AHF (*n* = 3913, 39%), 5.1% died in-hospital; (*Figure 1* shows percentages for HFrEF, HFmrEF, and HFpEF separately). The median (IQR) duration of hospitalization was 9 (6–14) days overall, 8 (4–18) days for those who died in hospital, and 9 (6–13) days for those discharged alive.

### Mortality and CV mortality over 1-year follow-up

Among patients with AHF, 36% survived to discharge but were lost to follow-up, and 59% had complete follow-up. Among patients with out-patient HF, 26% were lost to follow-up and 74% had complete follow-up. Among patients with complete follow-up (i.e. did not die in hospital and were not lost to follow-up), the follow-up time was 376 (360–432 days), and overall, 11% died (after AHF discharge: 20%; after out-patient visit: 6.2%).

**Figure 2:**
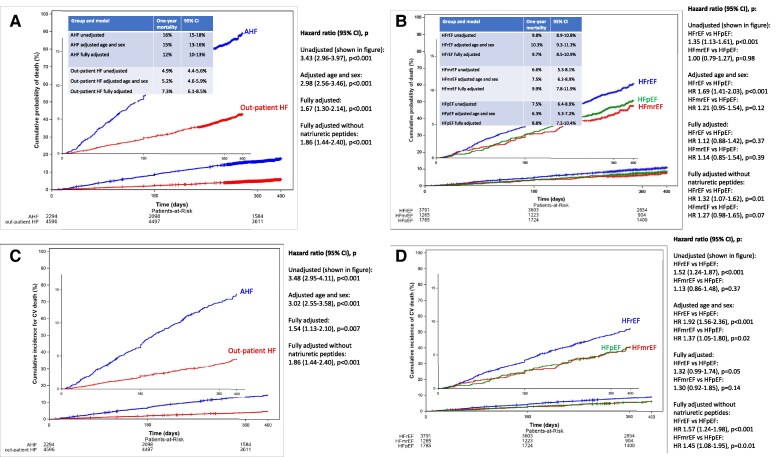
**A: All-cause mortality in AHF vs out-patient HF (all EF categories combined).** Patients lost to follow-up were excluded. In AHF, patients who died in hospital were excluded, and survival time is counted from time of hospital admission (baseline). Censoring occurred at last follow-up alive. The curves show cumulative incidence of all-cause death, unadjusted. The insert represents the same curves on a different scale. The inserted table shows percent 1-year mortality. Hazard ratios unadjusted include all patients not lost to follow up. Hazard ratios adjusted age and sex include age, sex and the age × sex interaction term. Hazard ratios fully adjusted are after adjustments for 14 covariates: age, sex, EF as continuous variable, previous hospitalization for HF, BMI, NYHA class (four levels), ischaemic aetiology yes/no, diabetes yes/no, systolic blood pressure, heart rate, number of symptoms (0–4 of rales, oedema, dyspnea at rest, orthopnea), haemoglobin, eGFR, natriuretic peptides; patients with missing data on any of these variables were excluded. A sensitivity analysis was performed adjusting for the same covariates except natriuretic peptides. 2B: All-cause mortality in HFrEF vs HFmrEF vs HFpEF (AHF and out-patient HF combined). Methods are as in *Figure 2A* (expect adjustment is for AHF vs out-patient status rather than EF). 2C: Cumulative incidence curve for CV death in AHF vs out-patient HF (all EF categories combined). Unknown cause of death was counted as CV death. Censoring occurred at non-CV death or last follow-up alive. Methods are as in *Figure 2A* except models are cumulative incidence functions rather than Cox models. 2D: Cumulative incidence curve for CV death in HFrEF vs HFmrEF vs HFpEF (AHF and out-patient HF combined). Methods are as in *Figure 2C*

For all-cause mortality and CV death, cumulative incidence curves, 1-year percent death/CV death, and HRs (95% CI) in unadjusted and adjusted analyses for different comparisons and different outcomes are shown in *Figure 2*. Overall survival was distinctly worse after discharge from AHF vs after an out-patient HF visit (*Figure 2A*). This difference was about half after full adjustment. Overall survival was significantly worse in HFrEF vs HFpEF in unadjusted analysis but this was no longer significant after full adjustment. Overall survival in HFmrEF vs HFpEF was no different in unadjusted analysis but nominally worse in HFmrEF after adjustment (*Figure 2B*).

For CV death, the HRs for AHF vs out-patient HF were roughly similar as for all-cause death, i.e. AHF vs out-patient HF was associated with a similar excess risk (∼2-fold) of both all-cause death and CV death (*Figure 2C*). Comparing EF categories, the excess risk with HFrEF was greater for CV death than for all-cause death. Unadjusted risk was greater for HFrEF vs HFpEF, but not for HFmrEF vs HFpEF. After adjusting for covariates, HFrEF vs HFpEF remained significantly or near significantly associated with risk of CV death, and HFmrEF vs HFpEF became significantly or near significantly associated with increased risk (*Figure 2D*).

### Event rates for mortality and causes of death and for total HF hospitalizations

**Figure 3:**
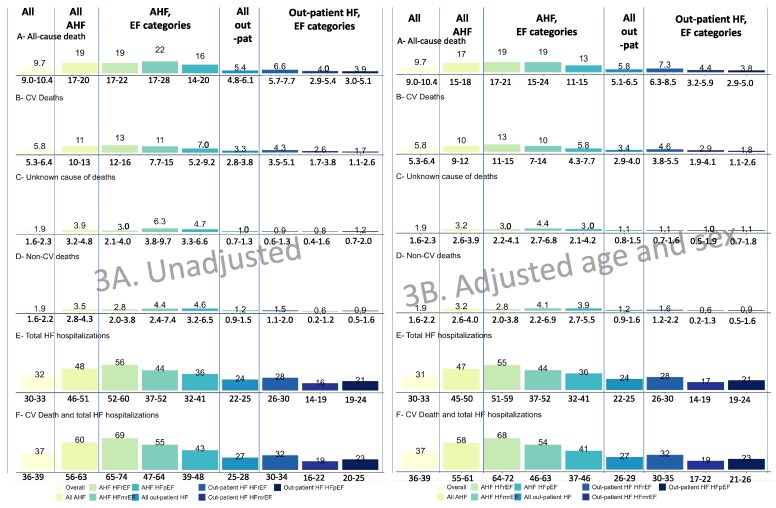
Event rates (95% CI) per 100 patient-years for numerous relevant outcomes in different combinations of EF category and AHF vs out-patient HF. Numbers are number of events per 100 patient-years of follow-up and ranges are 95% CIs. Patients with AHF who died during hospitalization and patients lost to follow-up are excluded. For patients with AHF who survived discharge, time is counted from day of admission (baseline). *Figure 3A* shows unadjusted event rates and *Figure 3B* shows event rates adjusted for age and sex

*Figures 3A* provides unadjusted event rates with 95% CIs per 100 patient-years of follow-up for numerous relevant outcomes. Rates of death from any cause per 100 patient-years of follow-up ranged from 3.9 to 22 depending on AHF vs out-patient status and EF category. CV death was ∼3-fold more common than non-CV death, and more dominant with lower EF. Unknown cause of death was relatively common, and if unknown cause of death were assigned to CV causes, then CV causes of death would be ∼4-fold more common than non-CV. Rates of total HF hospitalizations ranged from 16 to 56 and rates of total HF hospitalizations or CV death ranged from 19 to 69 per 100 patient-years, depending on AHF vs out-patient status and EF category. *Figure 3B* provides the corresponding event rates adjusted for age and sex.

### Number of HF hospitalizations and dispersion

**Figure 4:**
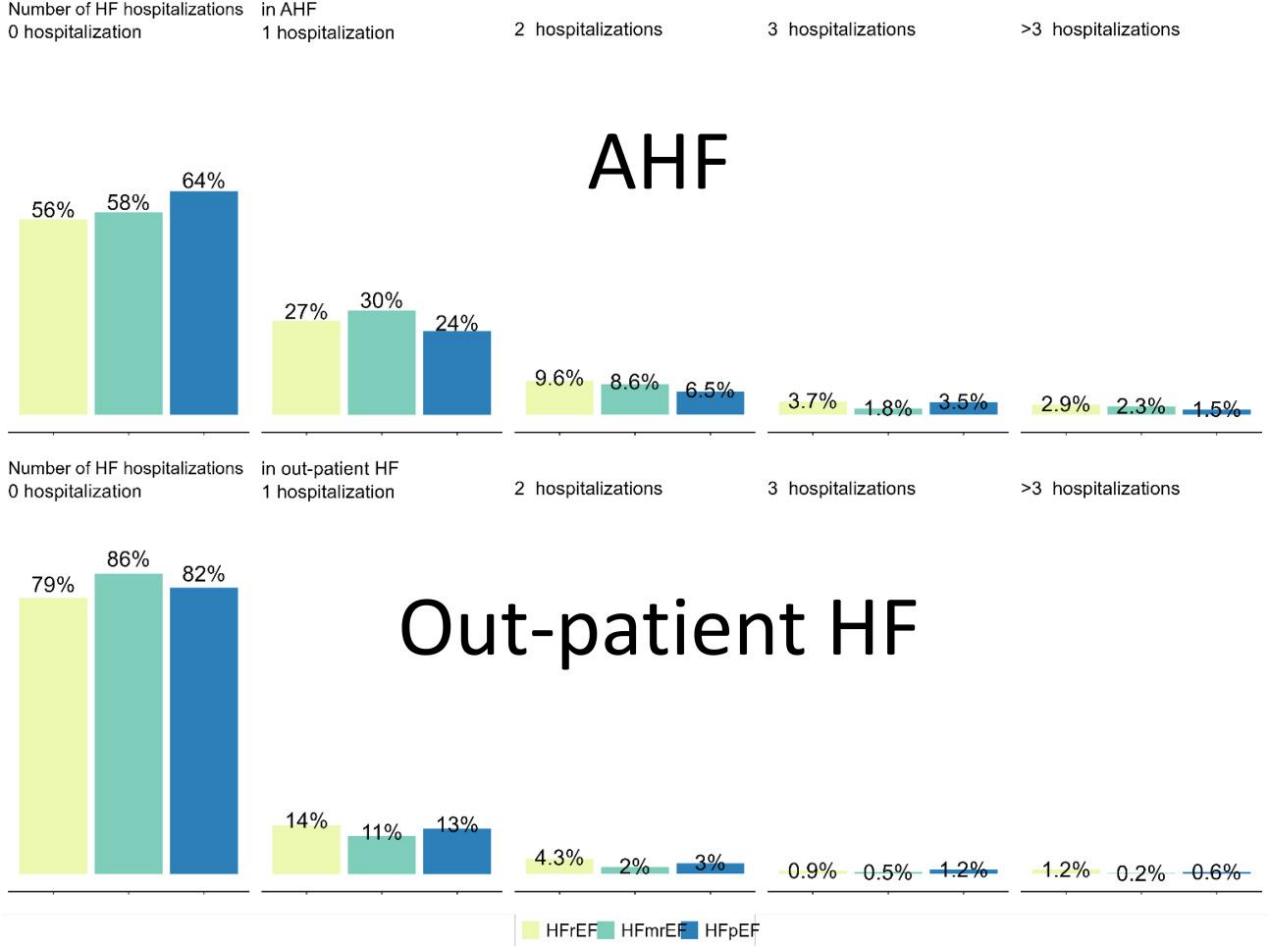
**Number of HF hospitalizations and dispersion**. Percentages are the percentage of patients who experienced 0, 1, 2, 3 or >3 hospitalizations for HF during a median follow up of 376 (360–432) days. Patients with AHF who died during the AHF hospitalization and patients lost to follow-up are excluded. For patients with AHF who survived to discharge, time is counted from day of admission (baseline) and number of hospitalizations exclude the baseline hospitalization

Overall, about two-thirds of patients did not experience a (re-)hospitalization for HF during the 1 year of -follow-up. The proportion of patients who experienced zero, 1, 2, 3, or >3 HF (re-)hospitalizations are depicted in *Figure 4*. At least one (re-)hospitalization for HF was experienced in 44% AHF HFrEF, 42% AHF HFmrEF, 36% AHF HFpEF, 21% out-patient HFrEF, 14% out-patient HFmrEF, and 18% out-patient HFpEF.

## Discussion

Characteristics, treatment, and outcomes of patients with HF have been assessed in multiple epidemiological, registry-based, and cohort-based analyses and trial datasets. However, each of these are limited by lack of clinical data (internal validity), generalizability (external validity), and/or comprehensiveness (e.g. did not include acute and out-patient HF, all EF categories, chronic and new-onset HF, and multiple variable domains [demographics, clinical, laboratory, imaging, therapy, and cause specific outcomes]). The present co-primary analysis describing outcomes in over 10 000 patients of the ESC HF III Registry meets all these criteria. In-hospital mortality was 5.1% (and greater with lower EF). Among hospital survivors and out-patients over 1 year of follow-up, event rates per 100 patient-years varied for death, 3.9–22, CV death 1.7–13, and unknown cause of death 0.8–6.3. The percent of patients that were (re-)hospitalized for HF at least once over 1-year follow-up ranged 14–44% and was twice as high post-AHF compared with post-out-patient visit (*Structured Graphical Abstract*).

### In-hospital outcomes in AHF

In AHF, in-hospital mortality was 3.4–5.2%, greater with lower EF. This was similar to the 4% in the US ADHERE Registry from 2003,^13^ and, notably, worse than the 2.2–3.4% in the previous ESC HF Registry,^14^ with a range 2–12%, higher with more comorbidities^15^ and worse congestion/perfusion status.^16^ AHF continues to lack evidence-based therapy, and it is possible that patients admitted to hospital have more severe HF in the recent era, when many patients with worsening HF who in the past might have been hospitalized are now managed with intravenous therapy but in the ambulatory setting.^17^ HF trials more recently have included patients with AHF but only after they have been stabilized in hospital and generally after transitioning to oral diuretics.^18^ Thus, new interventions and trials in patients with AHF still needing acute, intravenous treatment are sorely needed.

### One-year outcomes after AHF discharge and out-patient HF visit

The incidence of HF is decreasing, likely because of improved recognition and management of risk factors.^4^ However, the prevalence is increasing,^4^ and there have been concerns that outcomes in HF are not improving, especially in the USA,^19^ despite improved evidence-based therapy. In recent years, outcomes do seem to be improving at least in Europe.^20,21^ In the present analysis, enrolling patients in 41 European or ESC affiliated countries between 2018 and 2020, the risk of HF hospitalization, CV death, and all-cause death remained considerable but was lower than in previous ESC HF registries.^14,22^ Outcomes are highly variable globally, with generally better outcomes in higher income countries.^17,23^

Patients discharged after AHF have a high risk of re-hospitalization and death. In the present analysis, the crude risk of HF re-hospitalization was twice as high compared with after an out-patient visit. The crude risk of both CV death and all-cause death was more than 3-fold after AHF discharge compared with after an out-patient visit. After multivariable adjustment, this risk was reduced to 50% higher, suggesting that the post discharge risk is explained by HF itself being more advanced or severe but also associated with other risk factors. After multivariable adjustment without natriuretic peptides, the risk was nearly 100% greater, suggesting that natriuretic peptides alone, as a marker of congestion and severity of HF, explain a substantial amount of the risk in HF.

### One-year outcomes in HFrEF vs HFmrEF vs HFpEF

Differences between EF categories continue to be debated.^24,25^ Although there is considerable overlap, most data suggest that HFmrEF is milder than but otherwise similar to HFrEF, where both are generally the result of primary myocardial injury.^3,26^ In contrast, HFpEF appears to be a different syndrome, secondary to comorbidity-driven systemic inflammation and progressive fibrosis and cardiomyocyte changes.^27^ Indeed, the 2021 ESC guidelines state that ‘patients with HFmrEF have, on average, features that are more similar to HFrEF than HFpEF’, and recommend considering neurohormonal antagonists and modulators in HFmrEF but not in HFpEF.^6^ Since 2021, SGLT2 inhibitors and the non-steroidal mineralocorticoid receptor antagonist finerenone have proven effective in both HFmrEF and HFpEF, perhaps because they target inflammation and fibrosis.

In the present analysis, as expected, HFrEF had greater risk of CV death, death, and HF (re-) hospitalization than HFmrEF and HFpEF, even after extensive adjustment, suggesting that low EF itself is not only a risk marker but also a risk factor. HFmrEF and HFpEF had similar risk in crude analysis, but after adjustment, HFmrEF had greater risk, again confirming that the lower EF, even if only mildly reduced, is a risk factor, and that risk in HFpEF is more driven by covariates which are ‘adjusted away’ in multivariable analyses.

### Event rates per 100 patient-years over 1-year follow-up

Observational clinical research commonly expresses outcomes with Kaplan-Meier curves with time to first event and percent risk. However, in order to more comprehensively quantify event rates, to compare different outcomes in different patient categories and sub-groups, and to perform power calculations for interventional trials, event rates per 100 patient-years are more useful. The present analysis is unique in its broad inclusion of patients from 41 European and ESC-affiliated countries, making it generalizable and suitable for estimating event rates for trialists planning future randomized controlled trials in Europe. Furthermore, it is unique in its concurrent and comparative assessments of both acute and out-patient HF, both *de novo* and pre-existing HF, and all three EF categories. Thus, this analysis may serve as reference material for what to expect when including different patient categories and studying different outcomes, although, despite various enrichment criteria, clinical trials end up enrolling patients with lower event rates than registries and cohorts, both because of formal exclusion criteria of patients with e.g. cancer, and because of conscious or un-conscious selection bias among investigators towards younger and healthier patients.^28^

The present analysis presents event rates for the different patient categories for six different CV and non-CV outcomes. A few observations are especially important: markers associated with greater event rates such as acute vs out-patient HF and lower EF were largely expected but were also generally associated with both CV and competing non-CV outcomes, with the exception of lower EF actually being associated with lower risk of non-CV death. It would be helpful but remains difficult to identify risk markers other than e.g. EF which are associated with HF and CV events but not with all-cause or even non-CV events. From CV deaths alone to the composite of total CV deaths and HF hospitalizations, and in the different patient categories, event rates had an extremely wide range, from 1 to 69 per 100-patient-years, suggesting the statistical power of trials will vary extensively based on selection criteria. The rate of CV death in out-patient HFpEF was only 1.7 per 100 patient-years, providing exceedingly low power to detect a treatment effect on CV death in out-patient HFpEF. The rate in out-patient HFrEF was also low, at 4.3 per 100 patient-years, suggesting that a treatment effect on CV death in a HFrEF clinical trial would require a substantial portion enriched for advanced HFrEF and/or post AHF. Rates of HF (re)-hospitalization on the other hand were quite high suggesting novel interventions can still have substantial and clinically meaningful effects.

### Limitations

The ESC HF III Registry has broad European and ESC-affiliated coverage but participating sites are cardiology oriented and may not be representative of HF in internal medicine, geriatrics or primary care settings. This is indeed suggested by the relatively young age of patients, better use of GDMT in this ESC HF III Registry^12^ than in other more generalizable cohorts and registries or general populations,^29,30^ and relatively low event rates compared with population-based cohorts, especially in HFpEF, where many patients with higher age and more comorbidities may not be seen in cardiology departments. Use of natriuretic peptides was low. There were no mandated diagnostics or treatments in the protocol. Most patients had chronic HF and thus already a diagnosis established. Although repeat natriuretic peptide testing is common, their main value is for diagnosis, and investigators may not have considered repeat measurements clinically necessary or indicated. Surprisingly many patients were lost to follow-up, which is unlikely to occur at random but more commonly in older, frailer patients with higher mortality. In contrast to in a randomized controlled trial, there were no mandatory regular follow-up visits. A visit, contact or attempted contact at 12 months was specified in the protocol, but may have been difficult in older patients and at referral centres who may not follow patients continuously. Thus, the present results may underestimate true event rates during follow-up, particularly in older patients with HFpEF. Outcome collection during follow-up included death, cause of death, and date of death, allowing time to event analyses, and number of hospitalizations and causes of hospitalization, but not time to hospitalization, precluding time to first HF (re-) hospitalization or to the commonly used time to composite of first CV death or HF hospitalization event.

## Conclusions

The present analysis combines granular data for clinical characterization and for multivariable adjustment when assessing outcomes, providing internal validity and reliability, with large sample size and broad European and ESC-affiliated coverage, providing external validity and generalizability. It reports both in-hospital and 1-year post-discharge outcomes in AHF, and 1-year outcomes in out-patient HF, includes both *de novo* and pre-existing HF, and reports multiple relevant cause-specific outcomes and comparisons among all three EF categories. These data are useful for patient care and future trials and for all stakeholders working to improve outcomes in patients with HF (*Table 2*).

**Table 2:** Potential uses of ESC HF III Registry data

Potential uses of ESC HF III Registry data	
Data	Potential use
Patient characteristics	Epidemiological studies Ejection fraction category comparisons Resource allocation Patient phenotyping, cluster analyses, machine learning, artificial intelligence Trial design
Treatment	Implementation science Resource allocation Cost-effectiveness studies Education and awareness Screening Registry-based randomized controlled trial infrastructure and design
Cause-specific outcomes	Epidemiological studies Resource allocation, targeting and prioritizing treatment Cost-effectiveness studies Ejection fraction category comparisons Patient phenotyping, cluster analyses, machine learning, artificial intelligence Competing risk analyses Trial design
Event rates	Trial design

## Data Availability

Direct access to the HF III Registry dataset is limited to the EORP HF III Data Management and Statistical Analysis teams. Country-specific datasets may be provided to the national cardiology societies upon request to EORP.

## APPENDIX 1. EORP Oversight Committee

2016–2018: A. Vahanian, FR (Chair); A. Budaj, PL; N. Dagres, DE; N. Danchin, FR; V. Delgado, NL; J. Emberson, GB; O. Friberg, SE; C.P. Gale, GB; G. Heyndrickx, BE; B. Iung, FR; S. James, SE; A.P. Kappetein, NL; A.P. Maggioni, IT; N. Maniadakis, GR; K.V. Nagy, HU; G. Parati, IT; A-S. Petronio, IT; M. Pietila, FI; E. Prescott, DK; F. Ruschitzka, CH; F. Van de Werf, BE; F. Weidinger, AT; U. Zeymer, DE.

2018–2020: C.P. Gale, GB (Chair); B. Beleslin, RS; A. Budaj, PL; O. Chioncel, RO; N. Dagres, DE; N. Danchin, FR; J. Emberson, GB; D. Erlinge, SE; M. Glikson, IL; A. Gray, GB; M. Kayikcioglu, TR; A.P. Maggioni, IT; K.V. Nagy, HU; A. Nedoshivin, RU; A-P. Petronio, IT; J.W. Roos-Hesselink,

NL; L. Wallentin, SE; U. Zeymer, DE.

2020–2022: B.A. Popescu, RO (Chair); D. Adlam, GB; A.L.P. Caforio, IT; D. Capodanno, IT; Ovidiu Chioncel, RO; M. Dweck, GB; D. Erlinge, SE; Laurent Fauchier, FR; Marek Gierlotka, PL; M. Glikson, IL; Tina Hansen, DK; J. Hausleiter, DE; B. Iung, FR; M. Kayikcioglu, TR; P. Ludman, GB; LH. Lund, SE; A.P. Maggioni, IT; Julien Magne, FR; S. Matskeplishvili, RU; B. Meder, DE; Julinda Mehilli, DE; K.V. Nagy, HU; A. Nedoshivin, RU; D. Neglia, IT; A.A. Pasquet, BE; Eva Prescott, DK; J.W. Roos-Hesselink, NL; F.J. Rossello, ES; S.M. Shaheen, EG; A. Torbica, IT.

2022–2024: B. Iung, FR (Chair); B. Popescu, RO (Past-Chairperson); D Adlam, UK; C Bouleti, FR; A Caforio, IT; D Capodanno, IT; L Fauchier, FR; M Gilard, FR; S James, SE; P Ludman, UK; J Magne, France; A Pasquet, BE; T Pilgrim, CH; J Rossello, ES; S Shaheen, EG; AvTimoteo, PO; A Torbica, IT; U Zeymer, DE

## APPENDIX 2. ESC HF III Registry Executive Committee (board members of the HFA of the ESC)

Lars H. Lund, SE (Chairperson); Marisa Crespo-Leiro, ES (Immediate Past Chair); Petar Seferovic, RS; Frank Ruschitzka, CH; Gerasimos Filippatos, GR; Alexandre Mebazaa, FR; Massimo Piepoli, IT; Andrew Coats, AU; Stefan Anker, DE; Theresa McDonagh, UK; Mitja Lainscak, SI; Giuseppe Rosano IT; Aldo Maggioni, IT (First Chair; EORP representative)

## APPENDIX 3. ESC HF III Registry Steering Committee (National Coordinators)

Lars Lund, SE (Registry Chair); Ahmed Bennis, MA; Andrejs Erglis, LV; Andrzej Gackowski, PL; Alena Kurlianskaya, BY; Amina Rakisheva, KZ; Alex Simms, GB; Barnabas Gellen, FR; Bela Merkely, HU; Candida Fonseca, PT; Daniela Cassar Demarco, MT; Duska Glavas, HR; Eva Goncalvesova, SK; Eleonora Vataman, MD; Elizabeta Srbinovska Kostovska, MK; Erkin Mirrakhimov, KG; Gani Bajraktari, XK; Grigorios Giamouzis, GR; Artan Goda, AL; Gulnaz Dadashova, AZ; Hamayak Sisakian, AM; Hadi Skouri, LB; Heli Tolppanen, FI; Israel Gotsman, IL; Jean Beissel, LU; Jelena Celutkiene, LT; Jose Manuel Garcia Pinilla, ES; Larisa Dizdarevic-Hudic, BA; Lars Lysgaard Gullestad, NO; Leonid Voronkov, UA; Magdy Abdelhamid, EG; Micha T. Maeder, CH; Mitja Lainscak, SI; Morten Schou, DK; Massimo Francesco Piepoli, IT; Marija Polovina, RS; Milos Taborsky,CZ; Mikheil Tsverava, GE; Naima Hammoudi, DZ; Ovidiu Chioncel, RO; Petra Van Pol, NL; Plamen Gatzov, BG; Belma Pojskic, BA; Petar M. Seferovic, RS; Rudolf Berger, AT; Stefan Stoerk, DE; Timur Abdullaev, UZ; Tiina Uuetoa, EE; Vassilis Barberis, CY; Vyacheslav Mareev, RU; Wachter Rolf, DE; Walter Droogne, BE; Yuksel Cavusoglu, TR; Zumreta Kušljugić, BA.

## APPENDIX 4. ESC HF III Registry Investigators

Algeria: Algiers: S. Benkhedda, D. Djermane, M. Baouni, F. Benouareth, K. Mouzaoui, N. Dahimene, S. Mansouri, F. Kerkouri, Algiers: A. Chibane, M. Djouhri, S. Benferhat, L. Talbi, Algiers: Y. Bouhouita-Guermech, R. Benkouar, A. Boudrifa, H. Elnaajer, K. Bouasria, O. Kassoul, Y. Tir, Algiers: D-E. Nibouche, A. Sik, A. Bounah, Algiers: N. Hammoudi, G. Sofiane, Algiers: M. Bouame, A. Sayah, E. Tebbache, Bejaia: A. Kachenoura, Beni Messous: F. Daimellah, Blida: M.T.C. Bouafia, M. Bouafia, N. Dammene Debbih, W. Takdemt, Armenia: Gyumri: T. Manukyan, Yerevan: L. Tumasyan, A. Chilingaryan, A. Stepanyan, L. Tunyan, Yerevan: H. Hayrapetyan, K. Azaryan, M. Tadevosyan, H. Poghosyan, Yerevan: H. Sisakian, G. Martirosyan, L. Sahakyan, M. Hovhannisyan, S. Pepoyan, Belarus: Minsk: A. Kurlianskaya, D. Salauyou, A. Kozyrava, O. Shatova, T. Troyanova-Shchutskaia, Belgium: Aalst: M. Vanderheyden, A. Moya, H. Batjoens, Brussels: A-C. Pouleur, O. Gurne, E. Tshilanda, F. Severino, Genk: W. Mullens, M. Dupont, S. Christiaens, C. Claes, S. Lenaerts, Kortrijk: D. Derthoo, M. Dumoulein, S. Naessens, I. Senesael, M. De Coninck, Leuven: W. Droogne, E. Sels, V. Servaes, Liege: P. Troisfontaines, E. Hoffer, M. Melissopoulou, D. Malmendier, M. Massoz, S. Jacquet, V. Dinraths, Bosnia and Herzegovina: Sarajevo: E. Begic, A. Mujakovic, A. Subo, A. Selimagic, F. Custovic, D. Mackic, E. Dzambasovic-Karaselimovic, A. Cehajic, A. Djozic, A. Biscevic-Obradovic, F. Kadic, E. Hrvat, I. Kurbasic, M. Tuce, Sarajevo: A. Durak-Nalbantic, A. Dzubur, E. Jahic, S. Sokolovic, Z. Gljiva Gogic, K. Aganovic, F. Zvizdic, A. Begic, N. Resic, E. Hodzic, M. Halilcevic, M. Vucijak Grgurevic, N. Hadžibegic, N. Sabanovic Bajramovic, S. Miseljic, Tuzla: E. Smajic, Z. Kušljugic, L. Dizdarevic-Hudic, K. Kovacevic, D. Loncar, M. Kovacevic, M. Selimovic, U. Pajic, I. Hudic, A. Avdic, A. Bijedic, A. Brkic, D. Mršic, E. Becirovic, I. Bijedic, Zenica: B. Pojskic, J. Djelmic, M. Ejubovic, L. Pojskic, E. Ramic, M. Sammak, H. Selimovic, E. Stimjanin, M. Sut, H. Torlak, I. Bisco, D. Bogicevic, A. Brkovic, Brazil: Caetanópolis: R. Salgado, A. Silva, B. Azeredo, D. De Souza, I. Couri, L. Salgado, Bulgaria: Plovdiv: M. Tokmakova, D. Jovanovska, Sofia: N. Runev, B. Stoimenov, E. Manov, R. Pancheva, V. Kolev, Sofia: V. Mincheva, V. Tsanova, O. Eftimova, I. Gruev, M. Mintchev, V. Stoyanovski, T. Petrusheva, Croatia: Opatija: V. Persic, M. Barisic, D. Raljevic, Split: D. Glavas, D. Milicic, J.A. Borovac, T. Zaninovic Jurjevic, J. Sikic, V. Persic, K. Pesek, Zabok: K. Pesek, S. Roginic, N. Borsic, Zagreb: J. Sikic, T. Friscic, Z. Planinic, Cyprus: Nicosia: T. Christodoulides, S. Kakoulli, Nicosia: V. Barberis, T. Michaelidou, Egypt: Assiut: H. Hasan-Ali, M. Abdel Ghany, A. Halim, S. Dimitry, Aswan: P.P.S. Selwanos, M. Hassan, Benha: A-S.M. Sabry, Beni Suef: Y.A. Abdelhady Ahmed, T. Osman, M. Eshak, Cairo: M. El Sayed, K. Menyawi, A. Roshdy, W. El Kilany, A. Abdeltawab, A.R. El Sayed, Cairo: O. Botrous, A. El Bagoury, Cairo: A. Samir, H. Kandil, M. Hosny, M. Abdelghany, M. Abdelhamid, M.K. Shokry, S. Essam, A. Kamal, Giza: H. Ammar, A. Kamel, A. Magdy, H. Elnesr, M. Amin, Mansoura: M.M. Yousif, A.H. Eladawy, M.F. Areed, M.H. Abdelnabi, A.G. Mahmoud, M.F. Abdelrahman, Port Said: A. Elbahry, Zagazig: A. Saad, M. Ali, Estonia: Tallinn: T. Uuetoa, S. Udalova, T. Anier, S. Viks, B. Veermäe, Finland: Helsinki: H. Tolppanen, K. Oksaharju, France: Bron: N. Mewton, A. Jobbé Duval, C. Boiteux, C. Bergerot, G. Baudry, L. Sebbag, M. Buisson, D. Mohamed-Said, F. El Harrane, M. Koenig, N. El-Jonhy, Créteil: A. Galat, D. Bodez, C. Chalard, F. Djelaili, S. Guendouz, S. Oghina, T. Damy, M. Idjellidaine, M. Kharoubi, N. Djefel, Y. Sellah, Douai: R. Pilato, Fort de France: J. Inamo, A. Monfort, N. Ozier-Lafontaine, Grenoble: D. Guijarro, T. Fourme, A. Monard, P. Bouheret, Grenoble: M. Salvat, A. Boignard, C. Augier, C. Casset, M. Maurin, E. Plan, D. Pollet, Kremlin-Bicêtre: P. Jourdain, E. Berthelot, M.T. Bailly, N. Hrynchyshyn, G. Leprun, Le Chesnay: B. Livarek, J-L. Georges, N. Baron, C. Charbonnel, J-B. Azowa, Le Mans: C. Bachelet, P. Cloitre, P. Poret, S. Braun, O. Zid, G. Kabalu, A. Denizet Mulocher, C. Bros, C. Dericbourg, C. Saint Andre, E. Lefebvre, J-F. Laine, G. Terrien, J-C. Amirault, D. Lecuyer, M. Rousseau, Paris: F. Beauvais, A. Cohen-Solal, D. Logeart, N. Bennacer, Poitiers: B. Gellen, Toulon: J-M. Tartiere, F. Challal, F. Fellini, R. Landes, A. Elkeurti, C. Genin, P. Armangau, V. Gardan, Toulouse: M. Galinier, O. Lairez, J. Roncalli, C. Biendel, C. Delmas, C. Delon, E. Cariou, P. Fournier, R. Itier, Greece: Athens: A. Karavidas, D. Balta, C. Mandila, E. Velaoras, I.N. Karavidas, Ioannina: L. Michalis, K. Naka, A. Bechlioulis, L. Lakkas, A. Rammos, I. Dimou, Thessaloniki: C. Karvounis, M-A. Bazbani, Hungary: Budapest: B. Merkely, A. Kosztin, Budapest: N. Nyolczas, D. Vagany, I. Juhasz, S. Papp, M. Szabo, E. Szogi, Hodmezovasarhely: A. Pálinkás, I. Szoko Csaszar, I. Juhasz Nagy, S. Rostásné Toth, Pecs: T. Habon, M. Rabai, R. Halmosi, R. Gal, L. Illes, S. Kovacsne Levang, Iraq: Baghdad: H. Ali Farhan, I.F. Yaseen, Israel: Haifa: E. Radzishevsky, Jerusalem: I. Gotsman, O. Ezra, Safed: T. Levinas, I. Nordkin, S. Benyamin, Tel Aviv: M. Laufer-Perel, A. Milwidsky, B. Sadeh, D. Wexler, O. Havakuk, Y. Arbel, M. Revivo, S. Sadon, Italy: Brescia: M. Metra, L. Italia, L. Rossi, Cassano Delle Murge: A. Passantino, R. Carbonara, C. Rizzo, R. La Gioia, Florence: M. Milli, F. Grossi, Foggia: N.D. Brunetti, A. Mallardi, M. Correale, L’Aquila: M. Penco, G. Patti, S. Romano, R. Petroni, E. Salustri, S. Minardi, Lumezzane: S. Scalvini, E. Zanelli, A. Olivares, Milan: P. Agostoni, M. Doldi, E. Salvioni, P. Gugliandolo, Milano: A. Frisinghelli, C. Calloni, Monserrato: C. Cadeddu Dessalvi, M. Deidda, A. Peccianti, D. Cocco, E. Garau, Naples: A. Cittadini, A. Salzano, F. Giallauria, G. Crisci, R. Lucci, A.M. Marra, V. Valente, R. D´assante, Naples: P. Perrone-Filardi, P. Gargiulo, S. Paolillo, L. Bardi, S. Dellaversana, Palermo: G. Novo, A. Ferlisi, D. Adorno, E. Bonni, E. Corrado, F. Sabatino, S. Novo, Piacenza: M.F. Piepoli, B. Matrone, G. Halasz, L. Moderato, V. Pelizzoni, Rome: M. Mancone, C. Miotti, E. Pagliaroli, M. Magnocavallo, San Vito Al Tagliamento: D. Pavan, T. Durat, V. D’Onofrio, A. Gardin, G. Ganci, F. Martinis, N. Pezzutto, S. Maier, Trieste: G. Sinagra, M. Merlo, L. Restivo, F. Ramani, Udine: D. Miani, M. Baldassi, M. Driussi, Verona: F.L. Ribichini, L. Maritan, M. Cicoira, M. Lia, M. Setti, M. De Stefano, Kazakhstan: Aktobe: B. Zholdin, G. Mamedova, R. Iznatova, Y. Sim, Almaty: R. Tuleutayev, A. Nurmukhambetova, I. Yakupova, D. Urazbekov, Almaty: N. Aidargaliyeva, A. Akanova, A. Makhmudova, A. Tleules, G. Nurbekova, M. Akilbekova, S. Nurlybai, Almaty: A. Aizhan, Almaty: A. Rakisheva, A. Illyassova, Nur-Sultan: A. Dzholdasbekova, Z. Ismagulova, A. Kassenova, M. Alkenova, A. Aizhan, Kyrgyzstan: Bishkek: E. Mirrakhimov, K. Neronova, J. Esenbekova, Lithuania: Kaunas: D. Zaliaduonyte, J. Laukaitiene, J. Borkyte, A. Mazutaviciute, R. Norvilaite, Vilnius: J. Celutkiene, E. Paleviciute, J. Simonavicius, L. Gedvilaite, M. Laukyte, E. Lycholip, T. Simbelyte, Malta: Msida: R.G. Xuereb, S. Xuereb, W. Camilleri, M. Farrugia, J. Fleri Soler, L-L. Buttigieg, R. Xuereb, A.M. Moore, D. Cassar Demarco, C. Attard, M. Vella, I. Grech, R. Bonnici, R. Buhagiar, G. Buhagiar, T. Farrugia, Mexico: Culiacan, Sinaloa: M.O. De Los Rios Ibarra, A.Z. Baños Velasco, F.M. Vizcaíno Rios, Mexico: A. Mendez, A. Alvarez, C. Guizar, Queretaro: S. Leon Gonzalez, L. Resendiz-Barron, Moldova, Republic Of: Chisinau: E. Vataman, D. Bursacovschi, D. Lisii, E. Paraniuc, J. Cazacu, M. Dogot, N. Botnari, Chisinau: L. Grib, S. Filimon, E. Samohvalov, L. Purteanu, Chisinau: A. Grivenco, Chisinau: V. Revenco, I. Cabac-Pogorevici, I. Cojuhari, Morocco: Casablanca: A. Bennis, S. Chraibi, A. El Makhlouf, S. Ferhi, S. Soulami, N. Mouine, A. Moustaghfir, K. Adnan, C. Bencheqroun, Casablanca: G. Abidi, H. Elmousalami, S. Hajib, L. Bouzoubaa, M. Ghita, I. Nassiri, S. Abderrazak, Z. Benchaouia, G. Benhayoune, N. Boughaidi, D. Ghellab, Kenitra: A. Sourat, B. Menebhi, M. Bensaoud, A. El Ouazzani, A. Lachhab, A. Zakaria, F. El Idrani, H. Lachhab, Oujda: N. Elouafi, A. Hbali, G. Elmazani, I. Alla, Rabat: S. Ztot, M. Najat, B.E. El Younassi, I. Asfalou, L. Chami, M. Raissouni, Rabat: S. Abdelali, A. Benthami, A. Drissi Kacemi, S. Essadki, A. Louali, K. Bennis, Y. Lididi, R. Bouhouch, R. Mesbahi, S. Bouzidi Belmajdoub, Nigeria: Calabar: I. Ukpeh, R. Basake, E-O. Akpan, E. Emmanuel, O. Mbang, North Macedonia: Skopje: M. Gjerakaroska Radovikj, Skopje: E. Srbinovska Kostovska, I. Mitevska Peovska, L. Poposka, Norway: Lillehammer: G. Hogalmen, M. German, Oslo: L. Lysgaard Gullestad, C. Holt Bendz, O. Haugene, E. Bjorkelund, Poland: Katowice: K. Mizia-Stec, K. Bula, Krakow: J. Nessler, A. Gackowski, A. Siniarski, M. Kabat, Krakow: P. Rubis, A. Karabinowska, E. Dziewiecka, S. Wisniowska-Smialek, Lodz: M. Lelonek, A. Cieslak, A. Klaus, J. Szulc, K. Drazek, K.A. Nguyen, M. Kiedrowska, Lodz: A. Bielecka-Dabrowa, M. Pyziak Stepien, M. Rybak, P. Leczycki, P. Chrusciel, A. Wittczak, M. Banach, A. Chuda, A. Szwedzinska, M. Rembek Wieliczko, D. Mrozowska, F. Pawliczak, J. Lewek, M. Maciejewski, A. Cichocka-Radwan, A. Bikiewicz, K. Janikowski, Lublin: J. Szponar, A. Kujawa, A. Sutkowska, B. Cebulak, K. Pirog, J. Wieczorek, M. Suchecka, S. Goliszek, Opole: M. Gierlotka, E. Cierpiala, J. Bugajski, J. Plonka, K. Rekucki, Poznan: E. Straburzynska-Migaj, D. Budzynski, M. Gasiorowski, M. Dudek, Torun: G. Skonieczny, A. Dolacinska, A. Metzgier Gumiela, A. Stawicka, M. Rolirad, Warsaw: A. Kaplon-Cieslicka, M. Wawrzacz, K. Ozieranski, M. Kleszczewska, A. Tyminska, Warsaw: R.J. Gil, A. Pawlak, M. Wozniak, M. Pietraszek, A. Nazaruk, A. Bobel, A. Smolarczyk, D. Wiligorska, D. Ziecik, J. Latek, K. Byczkowska, Warszaw: P. Leszek, J. Urbanowski, N. Wiligorska, Zabrze: Z. Kalarus, T. Kukulski, A. Swiatkowski, M. Szulik, N. Kandora, Zielona Gora: P. Jesionowski, T. Zemleduch, A. Kasperowicz, K. Sosinska, Portugal: Faro: A. Camacho, R. Fernandes, H. Costa, Lisbon: C. Tavares Aguiar, A. Tralhão, A. Ventosa, C. Brízido, C. Strong, B. Rocha, G. Cunha, Lisbon: D. Brito, A. Nunes-Ferreira, I. Aguiar Ricardo, R. Santos, S. Pereira, T. Rodrigues, J. Agostinho, J. Brito, J. Rigueira, N. Cunha, P. António, P. Morais, D. Cravo, Lisbon: C. Fonseca, C. Rodrigues, I. Araujo, J. Presume, M. Proenca, S. Maltes, Porto: B. Moura, E. Barreira, P. Janeira, C. Ferrao, Porto: J. Silva-Cardoso, A. Sousa, B. Mena, X. Resende, M. Fonseca, M. Braga, M. Campelo, E. Moreira, P. Araújo, P. Diogo, R. Pinto, S. Amorim, B. Moura, Romania: Baia Mare: C. Pop, A. Iosip, Bucharest: C-J. Sinescu, N. Avram, D.G. Baltag, N. Samoila, Bucharest: M. Dorobantu, A. Scafa Udriste, A. Scarlatescu, N. Oprescu, O. Fronea Gheorghe, Bucharest: O. Chioncel, L. Antohi, O. Geavlete, Bucharest: G-A. Dan, I-C. Daha, S. Bari, C. Delcea, D.C. Ciuculete, I. Lupasteanu, C. Stanescu, A. Vijan, Craiova: C. Militaru, A. Giuca, G.C. Moise, E.G. Pascu, Galati: L. Grigorica, C. Corciova, Targu Mures: T. Benedek, M. Chitu, N. Rat, R. Hodas, D. Opincariu, I.S. Benedek, Timisoara: D. Lighezan, A.C. Florea, R. Buzas, V. Ivan, Russian Federation: Elektrostal: A. Molchanova, M. Piskareva, E. Bulgakova, E. Kudryashova, Kazan: A. Galyavich, L. Baleeva, Z. Galeeva, Krasnodar: A. Fendrikova, V. Skibitskiy, Z. Sokaeva, V. Babayan, N. Spiropulos, Moscow: E.V. Resnik, A.I. Kovaleva, M.S. Komissarova, V.A. Lazarev, Moscow: V. Larina, Moscow: Y. Belenkov, Y. Danilogorskaya, E. Zheleznykh, E. Privalova, I. Ilgisonis, V. Kaplunova, M. Kozhevnikova, G. Shakaryants, A. Shchendrygina, A. Yusupova, V. Zektser, Moscow: Z. Kobalava, E. Shavarova, L. Karapetyan, Moscow: G. Gendlin, A. Melekhov, I. Zakharova, M. Kuznetsova, M. Yunyaeva, Moscow: G. Arutyunov, A. Arutyunov, R. Muradyan, Moscow: S. Golitsyn, E. Gupalo, N. Mironova, Moscow: O. Drapkina, R. Myasnikov, T. Pavlunina, V. Dindikova, Y. Mareev, E. Andreenko, K. Krupychka, M. Kudryavtseva, O. Kulikova, Moscow: I. Orlova, J. Begrambekova, Nizhny Novgorod: N. Vinogradova, I. Fomin, Perm: N. Koziolova, A. Veclich, P. Karavaev, Rostov-On-Don: A. Chesnikova, O. Kolomatskaya, V. Gavrina, Ryazan: E. Smirnova, A. Karakiyan, I. Budanova, Saint-Petersburg: M. Sitnikova, A. Kuular, M. Trukshina, T. Lelyavina, Samara: D. Duplyakov, E. Zorina, R. Chernitsov, A. Sergeeva, Tomsk: A. Garganeeva, O. Tukish, K. Kopeva, V. Aleksandrenko, Ulyanovsk: A. Surminova, A. Tsareva, Vladivostok: T. Musurok, A. Garkusha, Serbia: Belgrade: A.D. Ristic, B. Ivanovic, D. Simic, M. Ašanin, P.M. Seferovic, J. Simic, V. Kovacevic, G. Krljanac, D. Matic, M. Mihailovic, M. Ostojic, M. Polovina, M. Tesic, D. Djikic, D. Simeunovic, I. Petrovic Djordjevic, I. Veljic, I. Milinkovic, K. Andjelkovic, A. Uscumlic, D. Sacic, Belgrade: M. Dekleva Manojlovic, S. Veljkovic, D. Stefanovic, J. Stevic, Belgrade: S. Hinic, M. Zdravkovic, A. Dokovic, J. Djokic, V. Mudrenovic, V. Popadic, S. Klasnja, Belgrade: S. Radovanovic, M. Radovanovic, V. Celic, A. Ilic, N. Blagojevic, D. Bosnjakovic, D. Toncev, Niš: S. Apostolovic, D. Stanojevic, S. Milutinovic, Niska Banja: B. Ilic, M. Deljanin Ilic, L. Nikolic, M. Stojanovic, D. Petrovic, D. Simonovic, S. Šaric, D. Hristov, Sremska Kamenica: I. Srdanovic, J. Dejanovic, T. Popov, S. Cemerlic Maksimovic, S. Dimic, S. Keca, V. Drljavic, D. Bogdanovic, I. Popov, J. Pavic Poljak, Slovakia: Bratislava: E. Goncalvesova, M. Luknár, Bratislava: P. Solik, Svidnik: M. Pytliak, P. Bojcík, Slovenia: Murska Sobota: M. Lainscak, N. Cmor, E. Dora, L. Majc Hodoscek, A. Vogrincic Cernezel, Trbovlje: B. Leskovar, T. Furlan, A. Milanovic, K. Vrbinc Vrtek, S. Poznic, V. Grilj, M. Režun, Spain: Alcazar de San Juan: V. Martinez Mateo, M.J. Fernandez Anguita, Cáceres: C. Ortiz Cortés, Denia: A. Valle Muñoz, H. Morillas Climent, J. Seller Moya, Figueres: S. Darnés Soler, S. Cudini, Granada: S. López Fernández, L. Jordán Martínez, F. Bermúdez Jiménez, La Coruna: M. Crespo Leiro, D. Couto Mallon, E. Barge Caballero, G. Barge Caballero, M.J. Paniagua Martin, P. Pardo Martinez, C. Naya Leira, C. Riveiro Rodriguez, M. Martinez Castro, P. Blanco Canosa, Z. Grille Cancela, Malaga: J.M. Garcia Pinilla, A. Robles Mezcua, A. Rodriguez Cordoba, C. Cruzado Alvarez, L. Morcillo Hidalgo, P. Marquez Camas, A.I. Perez Cabeza, P. Redondo-Gomez, M. Robles Mezcua, Ubeda: J.L. Bonilla Palomas, Sweden: Stockholm: L. Lund, M. Nygren, G. Savarese, C. Hage, E. Jonsson, E. Ottenblad, F. Granstrom, H. Lundberg, K. Karlsson, Syrian Arab Republic: Damascus: Y. Bani Marjeh, A. Abdin, F. Alhussein, F. Mgazeel, Turkey: Adana: F. Yavuz, Adiyaman: A. Karakus, Alanya Antalya: A. Coner, S. Akinci, Ankara: B. Demirkan, Antakya: O. Akkus, Antalya: A. Genc, Bursa: F.O. Arican Ozluk, Duzce: H. Harbalioglu, Eskisehir: Y. Cavusoglu, E. Babayigit, E. Sener, Gumushane: E.I. Yuce, Istanbul: H. Altay, Istanbul: Ö. Yildirimtürk, Izmir: C. Altin, Izmir: B. Kilicaslan, B. Unal, H. Acet, Izmir: N. Cetin, Kars: C. Burak, Kocaeli: D. Karacimen, Kocaeli: A. Agacdiken Agir, Y.U. Celikyurt, Mersin: A. Celik, E.E. Sahin, O. Sakarya, M. Demir, Mugla: O. Basaran, Samsun: A.E. Atas, Ukraine: Dnipro: O. Khaniukov, Ivano-Frankivsk: I. Vakaliuk, I. Drapchak, V. Sovtus, N. Tymochko, O. Prytuliak, Kharkiv: V. Tseluyko, N. Matviichuk, Kharkiv: M. Kopytsya, T. Storozhenko, Kharkiv: I. Rudyk, O. Medentseva, D. Babichev, Kiev: L. Voronkov, A. Liashenko, Kiev: I. Rudenko, K. Lazareva, N. Sishkina, A. Honcharuk, O. Vasylenko, Y. Antoniuk, Kiev: M. Dolzhenko, L. Hrubyak, L. Lobach, T. Simagina, Kiev: S. Kozhuhov, N. Dovganych, N. Thor, M. Danko, O. Yarynkina, O. Bazyka, Kiev: A. Parkhomenko, A. Stepura, D. Bilyi, O. Irkin, O. Dovhan, Kiev: V. Batushkin, D. Poddyachaya, Kiev: O. Zharinov, B. Todurov, I. Lischuk, Kiev: K. Rudenko, Vinnitsa: V. Zhebel, I. Pashkova, L. Sursaieva, Krivoy Rog: V. Potabashniy, V. Fesenko, O. Markova, O. Kniazieva, Zaporozhye: O. Berezin, O. Kremzer, United Kingdom: Bodelwyddan: M. Aldwaik, A. Bolger, R. Manley, V. Garvey, Uzbekistan: Tashkent: T. Abdullaev, S. Mirzarakhimova, A. Rasulov, A. Karimov, H. Gulomov, I. Tsoy, R. Kurbanova, R. Bekbulatova, Tashkent: U. Kamilova, D. Tagaeva.

## References

[1] Becher PM, Lund LH, Coats AJS, Savarese G. An update on global epidemiology in heart failure. Eur Heart J 2022;43:3005–7. 10.1093/eurheartj/ehac248 35578978

[2] Rosano GMC, Seferovic P, Savarese G, Spoletini I, Lopatin Y, Gustafsson F, et al Impact analysis of heart failure across European countries: an ESC-HFA position paper. ESC Heart Fail 2022;9:2767–78. 10.1002/ehf2.14076 35869679 PMC9715845

[3] Savarese G, Stolfo D, Sinagra G, Lund LH. Heart failure with mid-range or mildly reduced ejection fraction. Nat Rev Cardiol 2022;19:100–16. 10.1038/s41569-021-00605-5 34489589 PMC8420965

[4] Savarese G, Becher PM, Lund LH, Seferovic P, Rosano GMC, Coats A. Global burden of heart failure: a comprehensive and updated review of epidemiology. Cardiovasc Res 2023;118:3272–87. 10.1093/cvr/cvac013 35150240

[5] Johansson I, Joseph P, Balasubramanian K, McMurray JJV, Lund LH, Ezekowitz JA, et al Health-related quality of life and mortality in heart failure: the global congestive heart failure study of 23 000 patients from 40 countries. Circulation 2021;143:2129–42. 10.1161/CIRCULATIONAHA.120.050850 33906372

[6] McDonagh TA, Metra M, Adamo M, Gardner RS, Baumbach A, Bohm M, et al 2021 ESC guidelines for the diagnosis and treatment of acute and chronic heart failure. Eur Heart J 2021;42:3599–726. 10.1093/eurheartj/ehab368 34447992

[7] McDonagh TA, Metra M, Adamo M, Gardner RS, Baumbach A, Bohm M, et al 2023 focused update of the 2021 ESC guidelines for the diagnosis and treatment of acute and chronic heart failure. Eur Heart J 2023;44:3627–39. 10.1093/eurheartj/ehad195 37622666

[8] Kitzman DW, Brubaker P, Morgan T, Haykowsky M, Hundley G, Kraus WE, et al Effect of caloric restriction or aerobic exercise training on peak oxygen consumption and quality of life in obese older patients with heart failure with preserved ejection fraction: a randomized clinical trial. JAMA 2016;315:36–46. 10.1001/jama.2015.17346 26746456 PMC4787295

[9] Butler J, Shah SJ, Petrie MC, Borlaug BA, Abildstrom SZ, Davies MJ, et al Semaglutide versus placebo in people with obesity-related heart failure with preserved ejection fraction: a pooled analysis of the STEP-HFpEF and STEP-HFpEF DM randomised trials. Lancet 2024;403:1635–48. 10.1016/S0140-6736(24)00469-0 38599221 PMC11317105

[10] Packer M, Zile MR, Kramer CM, Baum SJ, Litwin SE, Menon V, et al Tirzepatide for heart failure with preserved ejection fraction and obesity. N Engl J Med 2025;392:427–37. 10.1056/NEJMoa2410027 39555826

[11] Lund LH, Crespo-Leiro MG, Laroche C, Garcia-Pinilla JM, Bennis A, Vataman EB, et al Rationale and design of the ESC Heart Failure III Registry—implementation and discovery. Eur J Heart Fail 2023;25:2316–30. 10.1002/ejhf.3087 37990135

[12] Lund LH, Crespo-Leiro MG, Laroche C, Zaliaduonyte D, Saad AM, Fonseca C, et al Heart failure in Europe: guideline-directed medical therapy use and decision making in chronic and acute, pre-existing and de novo, heart failure with reduced, mildly reduced, and preserved ejection fraction—the ESC EORP Heart Failure III Registry. Eur J Heart Fail 2024;26:2487–501. 10.1002/ejhf.3445 39257278 PMC11683873

[13] Adams KF, Fonarow GC, Emerman CL, LeJemtel TH, Costanzo MR, Abraham WT, et al Characteristics and outcomes of patients hospitalized for heart failure in the United States: rationale, design, and preliminary observations from the first 100,000 cases in the Acute Decompensated Heart Failure National Registry (ADHERE). Am Heart J 2005;149:209–16. 10.1016/j.ahj.2004.08.005 15846257

[14] Kaplon-Cieslicka A, Benson L, Chioncel O, Crespo-Leiro MG, Coats AJS, Anker SD, et al A comprehensive characterization of acute heart failure with preserved versus mildly reduced versus reduced ejection fraction—insights from the ESC-HFA EORP Heart Failure Long-Term Registry. Eur J Heart Fail 2022;24:335–50. 10.1002/ejhf.2408 34962044

[15] Chioncel O, Benson L, Crespo-Leiro MG, Anker SD, Coats AJS, Filippatos G, et al Comprehensive characterization of non-cardiac comorbidities in acute heart failure: an analysis of ESC-HFA EURObservational Research Programme Heart Failure Long-Term Registry. Eur J Prev Cardiol 2023;30:1346–58. 10.1093/eurjpc/zwad151 37172316

[16] Chioncel O, Mebazaa A, Maggioni AP, Harjola VP, Rosano G, Laroche C, et al Acute heart failure congestion and perfusion status—impact of the clinical classification on in-hospital and long-term outcomes; insights from the ESC-EORP-HFA Heart Failure Long-Term Registry. Eur J Heart Fail 2019;21:1338–52. 10.1002/ejhf.1492 31127678

[17] Filippatos G, Angermann CE, Cleland JGF, Lam CSP, Dahlstrom U, Dickstein K, et al Global differences in characteristics, precipitants, and initial management of patients presenting with acute heart failure. JAMA Cardiol 2020;5:401–10. 10.1001/jamacardio.2019.5108 31913404 PMC6990673

[18] Ouwerkerk W, Tromp J, Cleland JGF, Angermann CE, Dahlstrom U, Ertl G, et al Association of time-to-intravenous furosemide with mortality in acute heart failure: data from REPORT-HF. Eur J Heart Fail 2023;25:43–51. 10.1002/ejhf.2708 36196060 PMC10099670

[19] Sayed A, Abramov D, Fonarow GC, Mamas MA, Kobo O, Butler J, et al Reversals in the decline of heart failure mortality in the US, 1999 to 2021. JAMA Cardiol 2024;9:585–9. 10.1001/jamacardio.2024.0615 38656398 PMC11044007

[20] Lindberg F, Benson L, Dahlstrom U, Lund LH, Savarese G. Trends in heart failure mortality in Sweden between 1997 and 2022. Eur J Heart Fail 2025;27:366–76. 10.1002/ejhf.3506 39463287 PMC11860728

[21] Garred CH, Malmborg M, Malik ME, Zahir D, Christensen DM, Arulmurugananthavadivel A, et al Age-specific mortality trends in heart failure over 25 years: a retrospective Danish nationwide cohort study. Lancet Healthy Longev 2024;5:e326–35. 10.1016/S2666-7568(24)00029-1 38705151

[22] Chioncel O, Lainscak M, Seferovic PM, Anker SD, Crespo-Leiro MG, Harjola VP, et al Epidemiology and one-year outcomes in patients with chronic heart failure and preserved, mid-range and reduced ejection fraction: an analysis of the ESC Heart Failure Long-Term Registry. Eur J Heart Fail 2017;19:1574–85. 10.1002/ejhf.813 28386917

[23] G-CHF Investigators; Joseph P, Roy A, Lonn E, Stork S, Floras J, et al Global variations in heart failure etiology, management, and outcomes. JAMA 2023;329:1650–61. 10.1001/jama.2023.5942 37191704 PMC10189564

[24] Lund LH, Pitt B, Metra M. Left ventricular ejection fraction as the primary heart failure phenotyping parameter. Eur J Heart Fail 2022;24:1158–61. 10.1002/ejhf.2576 35703027

[25] Lund LH, Vedin O, Savarese G. Is ejection fraction in heart failure a limitation or an opportunity? Eur J Heart Fail 2018;20:431–2. 10.1002/ejhf.1106 29333631

[26] Lund LH, Claggett B, Liu J, Lam CS, Jhund PS, Rosano GM, et al Heart failure with mid-range ejection fraction in CHARM: characteristics, outcomes and effect of candesartan across the entire ejection fraction spectrum. Eur J Heart Fail 2018;20:1230–9. 10.1002/ejhf.1149 29431256

[27] Hamo CE, DeJong C, Hartshorne-Evans N, Lund LH, Shah SJ, Solomon S, et al Heart failure with preserved ejection fraction. Nat Rev Dis Primers 2024;10:55. 10.1038/s41572-024-00540-y 39143132

[28] Kapelios CJ, Benson L, Crespo-Leiro MG, Anker SD, Coats AJS, Chioncel O, et al Participation in a clinical trial is associated with lower mortality but not lower risk of HF hospitalization in patients with heart failure: observations from the ESC EORP Heart Failure Long-Term Registry. Eur Heart J 2023;44:1526–9. 10.1093/eurheartj/ehad109 36879413 PMC10149529

[29] Savarese G, Carrero JJ, Pitt B, Anker SD, Rosano GMC, Dahlstrom U, et al Factors associated with underuse of mineralocorticoid receptor antagonists in heart failure with reduced ejection fraction: an analysis of 11 215 patients from the Swedish Heart Failure Registry. Eur J Heart Fail 2018;20:1326–34. 10.1002/ejhf.1182 29578280

[30] Lund LH, Carrero JJ, Farahmand B, Henriksson KM, Jonsson A, Jernberg T, et al Association between enrolment in a heart failure quality registry and subsequent mortality-a nationwide cohort study. Eur J Heart Fail 2017;19:1107–16. 10.1002/ejhf.762 28229520

